# TREM-1 as a novel immunotherapeutic target to treat pancreatic ductal adenocarcinoma

**DOI:** 10.1016/j.omton.2025.201034

**Published:** 2025-08-14

**Authors:** Lina Gross, Ivanina Mutisheva, Hanne Hillen, Steve Robatel, Martin Wartenberg, Feiyang Ma, Lukas Bäriswyl, Delphine J. Lee, Robert L. Modlin, Kaspar Z’graggen, Mirjam Schenk

**Affiliations:** 1 Christine Kühne – Center for Allergy Research and Education, 7265 Davos, Switzerland; 2 Graduate School Cellular and Biomedical Sciences, University of Bern, 3012 Bern, Switzerland; 3 Division of Experimental Pathology, Institute of Tissue Medicine and Pathology, University of Bern, 3008 Bern, Switzerland; 4 Division of Surgical Pathology, Institute of Tissue Medicine and Pathology, University of Bern, 3008 Bern, Switzerland; 5 Department of Cell and Developmental Biology, Feinberg School of Medicine, Northwestern University, Chicago, IL 60611, USA; 6 The Lundquist Institute for Biomedical Innovation, Torrance, CA 90502, USA; 7 Division of Dermatology, Department of Medicine, Harbor-UCLA Medical Center, Torrance, CA 90502, USA; 8 David Geffen School of Medicine, University of California, Los Angeles, Los Angeles, CA 90095, USA; 9 Division of Dermatology, School of Medicine, University of California, Los Angeles, Los Angeles, CA 90095, USA; 10 Swiss Pancreas Clinic, 3013 Bern, Switzerland

**Keywords:** immunotherapy, TREM-1 activation, PDAC, myeloid cells, macrophage reprogramming

## Abstract

Pancreatic ductal adenocarcinoma (PDAC), the most common type of pancreatic cancer, is highly aggressive with limited curative options, primarily surgical resection. However, only about 20% of the tumors are resectable at diagnosis. Immunotherapies have largely failed in PDAC due to its immunosuppressive tumor microenvironment (TME). This study explores the potential of triggering receptor expressed on myeloid cells 1 (TREM-1) activation in altering the TME and enhancing tumor immunity in PDAC. Using the Pan02 mouse model and single-cell RNA sequencing (scRNA-seq), we found that intra-tumoral TREM-1 activation significantly reduced Pan02 tumor growth, an effect absent in *Trem1*^−/−^ mice. Our findings indicate that TREM-1 activation shifts tumor-associated macrophages (TAMs) and tumor-associated neutrophils (TANs) toward a pro-inflammatory state, promoting antitumor immune responses. Additionally, we show that TREM-1^+^ myeloid cells infiltrate human PDAC tissue. These results suggest that TREM-1 activation could reprogram the immunosuppressive TME, offering a promising strategy for PDAC treatment.

## Graphical abstract

**Figure:**
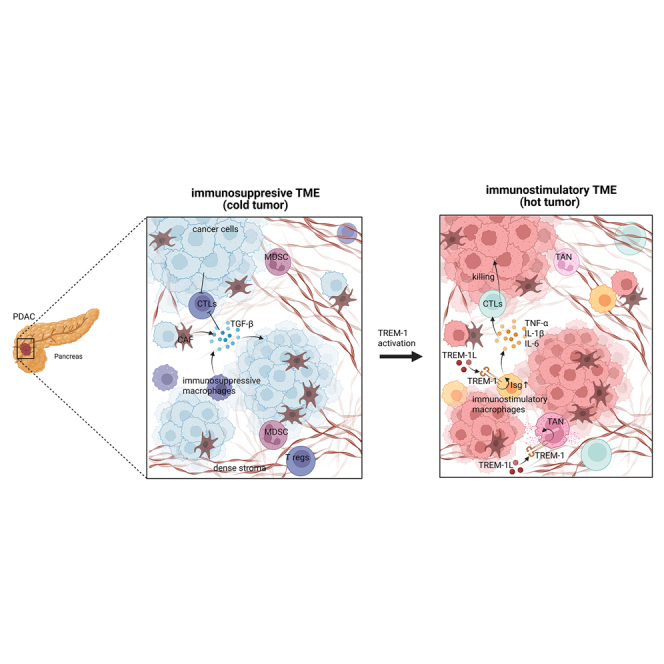

## Teaser

Pancreatic ductal adenocarcinoma (PDAC) is among the deadliest cancers, with limited treatment options and resistance to immunotherapy. We show that activating TREM-1 reprograms myeloid cells, transforms the tumor microenvironment, and boosts antitumor immunity, identifying TREM-1 as a promising immunotherapeutic target for treating PDAC.

## Introduction

Pancreatic ductal adenocarcinoma (PDAC) represents one of the most lethal diseases in economically developed countries and is the third leading cause of cancer deaths worldwide.^1^ The 5-year overall survival of PDAC patients is only about 7%, with incidence and mortality rates expected to increase over the next decades.^1^ Due to the non-specific symptoms such as weight loss and abdominal pain, the diagnosis is usually only made at an advanced stage, which further limits the treatment options.^2^^,^^3^ Several factors have been associated with an increased probability of developing PDAC, the most common of which are age,^4^^,^^5^ smoking,^6^ chronic pancreatitis due to excessive alcohol consumption,^7^^,^^8^ genetic predisposition,^6^ and family history.^9^

Surgery remains the only available treatment with curative intent but often leads to an average survival time of only about 8–10 months, due to the high relapse rate.^10^ It has been shown that the standard of care, adjuvant chemotherapy, extends survival time to around 26 months and increases the 5-year overall survival to about 30%.^11^^,^^12^ However, approximately 80% of PDAC cases are not eligible for surgery due to locally advanced stages and distant metastases^10^ and are therefore either subjected to neoadjuvant radio-/chemotherapy or palliative care. Neoadjuvant treatment can potentially result in downstaging and consequently render locally advanced tumors resectable in about one-third of the cases.^13^ Specifically, neoadjuvant chemotherapy can reduce tumor burden, enabling surgery and prolonging survival in some cases.^14^^,^^15^ Unfortunately, tumor progression is observed in most patients, even throughout chemotherapy cycles due to treatment resistance. Furthermore, immune checkpoint inhibitors such as anti-PD-1/PD-L1 and anti-CTLA-4, which have been shown to be efficient for the treatment of solid tumors such as breast cancer and melanoma, are ineffective in PDAC, partially due to its unique immunosuppressive tumor microenvironment (TME), low antigenicity, and mutational burden.^16^ Thus, there is an urgent need for novel treatments to improve the outcome of PDAC patients.

A major characteristic of PDAC is the desmoplasia, defined as the increased deposition of extracellular matrix by cancer-associated fibroblasts (CAFs). This dense stroma forms a physical barrier to infiltrating effector cells, impedes blood flow, and thus limits the delivery of therapeutic drugs within the tumor.^17^ Pancreatic stellate cells (PSCs) also contribute to the formation of desmoplasia and an immunosuppressive TME by secreting collagen, as well as various growth factors, including transforming growth factor β (TGF-β), platelet-derived growth factor (PDGF), and fibroblast growth factors (FGFs), thereby promoting tumor progression and metastasis.^18^^,^^19^ In addition, the immune compartment of PDAC is characterized by a substantial proportion of regulatory T cells (Tregs), myeloid-derived suppressor cells (MDSCs), and tumor-associated macrophages (TAMs), which further promote the immunosuppression.^20^ TAMs represent a major immune component of the TME in PDAC and contribute to immunosuppression in several ways. They secrete interleukin-10 (IL-10) and arginase, both suppressing T cell activity. They promote angiogenesis through the production of vascular endothelial growth factor (VEGF), thereby supplying the tumor with nutrients and oxygen, and they produce colony-stimulating factor 1 (CSF-1) to further recruit and sustain TAMs in the TME.^21^^,^^22^ Therefore, novel therapeutics targeting immunosuppression induced by myeloid cells hold great promises.

In recent years, there has been significant progress in understanding the roles of myeloid cells in the TME, with a focus on their dual capacity to either suppress or promote antitumor immunity. For example, Juric et al. demonstrated that the activation of triggering receptor expressed on myeloid cells 1(TREM-1) using the agonistic antibody PY159 could stimulate proinflammatory signaling pathways and enhance antitumor immunity in various cancer models, particularly through the reprogramming of TAMs and neutrophils into a proinflammatory state.^23^ In our study, we explore the potential of a ligand-based strategy to modulate myeloid cell activity within the pancreatic cancer TME, drawing parallels to the effects observed with TREM-1 activation but employing a different mechanism of action in another cancer type.

TREM-1 is a receptor expressed on macrophages, monocytes, and neutrophils that amplifies inflammatory responses, especially through synergism with Toll-like receptor (TLR) signaling.^24^^,^^25^ Possible ligands for TREM-1 are either endogenous danger signals released by necrotic cells, such as HMGB1 and Hsp70,^26^ or peptidoglycan (PGN) recognition protein 1 (PGLYRP1) complexed with PGN.^27^

TREM-1 activation triggers an oxidative burst,^28^^,^^29^ along with the release of pro-inflammatory cytokines, including IL-8 and myeloperoxidase (MPO), in neutrophils. In monocytes, TREM-1 activation leads to the secretion of IL-8, monocyte chemoattractant protein 1 (MCP-1), and tumor necrosis factor alpha (TNF-α).^30^^,^^31^^,^^32^ We hypothesize that TREM-1 activation can induce the repolarization of immunosuppressive myeloid cells toward a more inflammatory phenotype in the TME of PDAC.

## Results

### TREM-1-positive myeloid cells infiltrate human PDAC tissue, and TREM-1 expression positively correlates with classically activated macrophages

**Figure 1:**
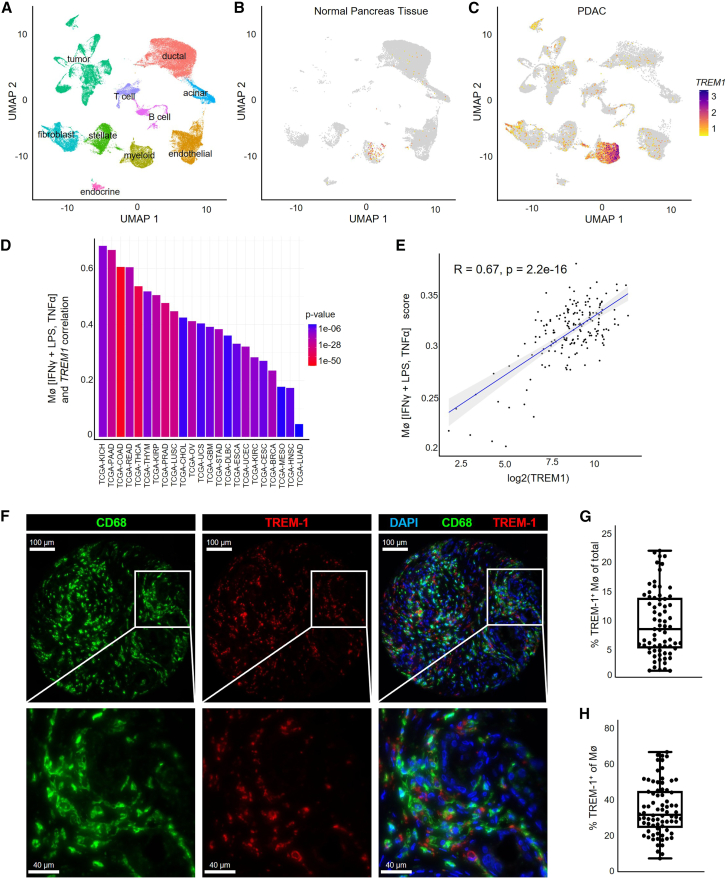
TREM-1^+^ myeloid cells infiltrate human PDAC tissue and positively correlates with M1-like signature (A) Cell-type identity and TREM1 expression in normal pancreatic tissue (*n* = 11) (B) compared to PDAC (*n* = 24) (C) in published single-cell RNA sequencing data.^33^ (D) Pearson correlation between *TREM1* gene expression and classically activated macrophage signature across TCGA cohorts. (E) Pearson correlation between *TREM1* expression and macrophage score in the TCGA-PAAD cohort. (F) Immunofluorescence (IF) images and magnification of human PDAC tissue stained for CD68 and TREM-1. Quantification of TREM-1^+^ cells as percentage of total cells (G) and percentage of macrophages (H) (*n* = 76).

Based on previous studies describing the presence of TAMs in PDAC, we reanalyzed published single-cell RNA sequencing (scRNA-seq) data from normal human pancreatic tissue and PDAC samples^33^ and identified 10 main clusters including fibroblast, ductal, acinar, endothelial, myeloid, endocrine, stellate, tumor, T, and B cells (Figures 1A, S1A, and S1B). Notably, a higher frequency of *TREM1*^+^ myeloid cells was observed in PDAC compared to control pancreatic tissue (Figures 1B and 1C). As TREM-1 is known to play a crucial role in the classical pathway of macrophage activation, we correlated *TREM1* expression with a signature for classically activated macrophages (interferon gamma [IFNγ] + lipopolysaccharide [LPS], TNF-α)^34^ within all TCGA cancer cohorts. This analysis identified the second highest correlation between *TREM1* gene expression and the classically activated macrophage signature in pancreatic adenocarcinoma (PAAD) (Figure 1D). Moreover, Pearson analysis revealed a strong positive correlation between *TREM1* gene expression and the macrophage signature in the PDAC cohort (Figure 1E). Next, we investigated TREM-1 protein expression in PDAC using immunofluorescence (IF) on formalin-fixed paraffin-embedded (FFPE) tissue samples of human PDAC patients (*n* = 76) (Figures 1F, S2A, and S2B). Therefore, we stained the tissue with DAPI, the myeloid cell marker CD68, and TREM-1. All PDAC samples had TREM-1-positive cells ranging from about 1% to over 20% of total cells (Figure 1G), and on average about one-third of all macrophages were TREM-1^+^ (Figure 1H). Taken together, these data demonstrate that TREM-1 is expressed on myeloid cells, which infiltrate PDAC, and that TREM-1 expression positively correlates with classically activated macrophage polarization.

### TREM-1 ligand induces the secretion of pro-inflammatory cytokines in human monocytes

**Figure 2:**
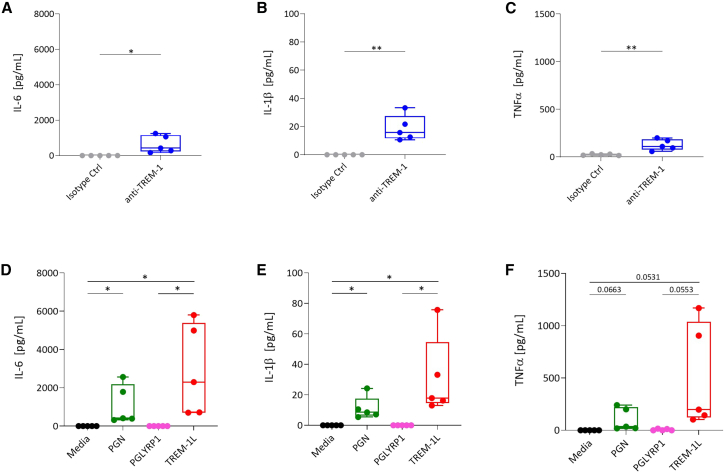
TREM-1L induces the secretion of pro-inflammatory cytokines more strongly than Ab-cross-linking of TREM-1 Human monocytes were stimulated with agonistic anti-TREM-1 or isotype control and cross-linked with a secondary antibody (A–C) or cultivated in media, supplemented with PGN, PGLYRP1, or TREM-1L (PGN-PGLYRP1) (D–F) for 24 h. Production of IL-6, IL-1β, and TNF-α was measured by ELISA. Statistical analysis was performed by two-tailed unpaired t test (*n* = 5 biological replicates). Significance: ∗*p* ≤ 0.05, ∗∗*p* ≤ 0.01, ∗∗∗*p* ≤ 0.001.

Previous studies have identified the peptidoglycan recognition protein 1 (PGLYRP1) complexed with PGN (PGN-PGLYRP1) as a TREM-1 ligand (TREM-1L) to activate myeloid cells.^27^ Therefore, we investigated the effect of TREM-1L (PGN-PGLYRP1) on TREM-1 activation in human monocytes and compared it to stimulation using an agonistic TREM-1 antibody followed by crosslinking. In both conditions, TREM-1 activation triggered the production of the pro-inflammatory cytokines IL-6, IL-1β, and TNF-α (Figures 2A–2F). The highest production of pro-inflammatory cytokines was induced by TREM-1L stimulation (Figures 2D–2F). In comparison, we observed no cell activation upon treatment with isotype control and crosslinking antibody (Figures 2A–2C), media, or PGLYRP1 alone (Figures 2D–2F). PGN stimulation triggered cytokine production potentially through TLR signaling.

Taken together, these results show that TREM-1 activation with TREM-1L in human monocytes induce a higher secretion of the pro-inflammatory cytokines IL-6, IL-1β, and TNF-α compared to TREM-1 activation via its agonistic antibody. Therefore, using this complex as a TREM-1 activator can exert more potent effects on triggering a pro-inflammatory immune response.

### TREM-1 activation reduces Pan02 tumor growth in mice

**Figure 3:**
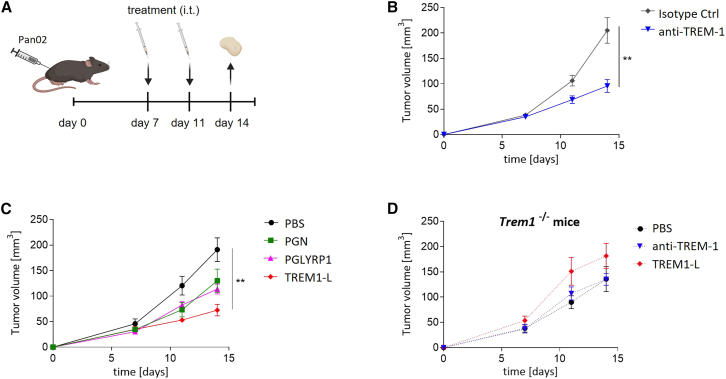
Intra-tumoral treatment with anti-TREM-1 Ab or TREM-1L reduced Pan02 tumor growth in mice (A) Experimental design: C57BL/6 were subcutaneously injected with Pan02 tumor cells. Treatments were administered intra-tumorally (i.t.) on days 7 and 11, followed by tumor collection on day 14. (B) Growth curve of Pan02 tumors treated with isotype control or anti-TREM-1 antibody (*n* = 12 per group). (C) Growth curve of Pan02 tumors treated with PBS, PGN, PGLYRP1, or TREM-1L (n = 5–6 per group). (D) Growth curve of Pan02 tumors in *Trem1*^−/−^ mice treated with PBS, anti-TREM-1 antibody, or TREM-1L (n = 6–9 per group). Data are represented as mean ± SEM using two-way ANOVA followed by Šidák’s multiple comparisons test. Significance: ∗*p* ≤ 0.05, ∗∗*p* ≤ 0.01, ∗∗∗*p* ≤ 0.001.

We assessed the effect of TREM-1 activation on tumor growth in the Pan02 mouse model of PDAC. Tumor-bearing mice were treated with intra-tumoral injections of anti-TREM-1 antibody or TREM-1L on days 7 and 11 post-tumor inoculation. Tumor growth was measured, and tumors were collected and analyzed on day 14 (Figure 3A). TREM-1 activation using the agonistic antibody significantly reduced tumor growth compared to isotype control (Figure 3B). However, treatment with TREM-1L was even more potent at reducing tumor growth (approximately 2-fold), while treatments with either PGN or PGLYRP1 alone had no significant effect (Figure 3C). We confirmed that the delayed tumor growth was TREM-1-dependent using *Trem1*^−/−^ mice. The beneficial effect of TREM-1 activation on tumor growth was abrogated with either treatment in the absence of TREM-1, confirming that the decrease in tumor volume is TREM-1 specific (Figure 3D).

Altogether, these results show that intra-tumoral TREM-1 activation reduces tumor growth in Pan02 tumors, indicating a potential use for TREM-1 activation to treat PDAC.

### scRNA-seq of Pan02 tumors reveals TREM-1 expression in TAN and TAM clusters

**Figure 4:**
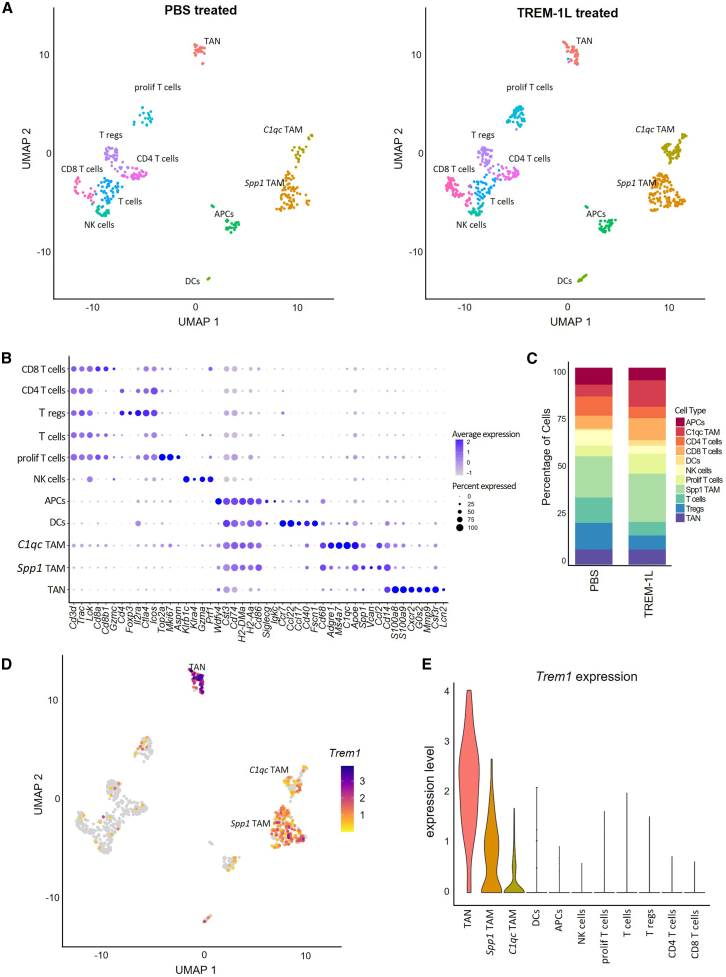
*Trem1* is highly expressed in TANs and TAMs in Pan02 tumors, and TREM-1 activation increases the proportion of effector cells, while reducing the number of regulatory T cells (A) UMAP plots demonstrating clustering and cell-type identities in Pan02 tumors treated with PBS (349 cells, 2 mice) or TREM-1L (641 cells, 2 mice). (B) Cell-type proportion of Pan02 tumors treated with PBS (gray) or TREM-1L (red). (C) Dot plot with cell-type-specific cluster marker. (D) Feature plot showing *Trem1* expression in TANs and TAMs. (E) Violin plots showing *Trem1* expression in TANs and TAMs.

Following the significant TREM-1-induced reduction of Pan02 tumor growth in mice, we investigated the cellular and transcriptomic changes induced by TREM-1 activation by performing scRNA-seq. We used low cell counts for sequencing (around 1,000 cells) with high sequencing depth (400–500 k reads per cell), which allowed us to detect neutrophils in our dataset. Neutrophils are short-lived cells, very sensitive to handling procedures and with particularly low mRNA levels, leading to their underrepresentation or absence in droplet-based scRNA-seq datasets.^35^ Unsupervised clustering followed by Uniform Manifold Approximation and Projection (UMAP) dimensionality reduction identified 11 distinct immune cell populations in the PBS and TREM-1L-treated Pan02 tumors (Figures 4A and S5A). The cell types included natural killer (NK) cells, CD4^+^ and CD8^+^ T cells, Tregs, proliferating T cells, dendritic cells (DCs), antigen-presenting cells (APCs), tumor-associated neutrophils (TANs), and two clusters of TAMs. One TAM cluster exhibited higher expression of *C1qc*, while the other was characterized by high *Spp1* expression, both representing distinct subtypes of TAMs, which cannot be distinguished based on M1/M2 signatures.^36^ Previous studies have linked *C1qc*^+^ TAMs to phagocytosis, antigen presentation, and anti-tumor immune response, while *Spp1*^+^ TAMs are often associated with immunosuppression.^36^ The APC cluster is heterogeneous, containing a mixture of macrophages, DCs, and some B cells. The less-defined T cell cluster represents a mixed cell population consisting of CD8^+^ T cells, NK cells, and innate lymphoid cells (ILCs). Cell-type annotation was supported by SingleR predictions and validated using established cell-type markers (Figures 4B and S5B). Comparison of cell-type distribution between treatment groups revealed compositional shifts upon TREM-1 activation. TREM-1L-treated tumors displayed an increased proportion of *C1qc*^+^ TAMs, CD8^+^ T cells, and proliferating T cells, alongside with a notable reduction in Tregs, as visualized in the UMAP split by treatment (Figure 4A) and quantified in the cell-type proportion plot (Figure 4C). These findings were validated using flow cytometry, which demonstrated elevated frequencies of CD4^+^ and CD8^+^ T cells, NK cells, and NK T cells in TREM-1L-treated tumors, along with increased CD69 expression, suggesting an activated state (Figure S4).

Next, we assessed the expression of *Trem1* and found the highest expression in TANs, followed by both clusters of TAMs, with higher levels in *Spp1*^+^ TAM (Figures 4D and 4E). Overall, these findings demonstrate that TREM-1 activation remodels innate and adaptive immune compartments in the Pan02 tumor, characterized by an expansion of activated CD8^+^ T cells while reducing immunosuppressive Tregs. Moreover, we show that *Trem1* is highly expressed in TANs and TAMs.

### TREM-1 activation induces major changes in myeloid cell populations

**Figure 5:**
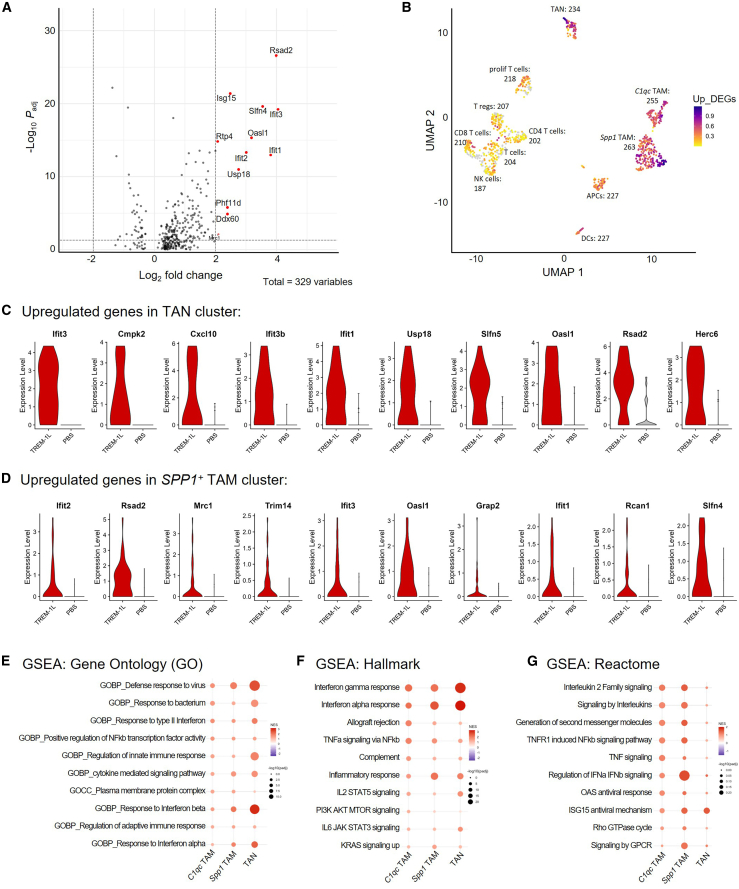
Differential gene expression analysis reveals major changes in myeloid populations upon TREM-1 activation (A) Upregulated genes following TREM-1L treatment in all cell clusters as identified by pseudobulk analysis. (B) Volcano plot showing average fold-change of up- (red) and downregulated (blue) pseudobulk genes based on their *p* value after TREM-1L treatment. (C) Top upregulated genes in TAN cluster. (D) Top upregulated genes in *Spp1*^*+*^ TAM cluster. (E–G) Pathway enrichment analysis with upregulated genes in TAN and *Spp1*^*+*^ TAM clusters after TREM-1L treatment.

To further investigate the changes induced by TREM-1 activation, we performed differential gene expression analysis. First, we identified up- and downregulated genes independent of cell type (pseudobulk), which are presented in a volcano plot using log_2_ fold change and adjusted *p* value (Figure 5A). This shows that there are more differentially expressed genes (DEGs) upregulated in TREML-treated tumors vs. PBS-treated tumors and highlights the most significantly different genes. Next, we determined which cell clusters express the majority of upregulated DEGs by visualizing them as a signature. As expected, the myeloid clusters expressing TREM-1, namely TAN, *Spp1*^*+*^ TAM, and *C1qc*^*+*^ TAM, expressed most of the upregulated DEGs upon TREM-1L treatment in Pan02 (Figure 5B).

Focusing on the two cell populations with the highest *Trem1* expression, we identified the top 10 upregulated genes within TANs (Figure 5C) and *Spp1*^+^ TAMs (Figure 5D). Consistent with the global DEG analysis, we found a prominent upregulation of IFN-associated genes in both populations, including *Rsad2*, *Slfn4*, *Oasl1*, *Oasl2*, *Ifit1*, *Ifit2*, and *Ifit3*. Moreover, TANs upregulate *Cxcl10*, also known as IFNγ-induced protein 10 (IP-10), a chemokine that recruits T cells, NK cells, and monocytes to sites of inflammation, whereas *Spp1*^+^ TAMs upregulate *Cxcl2* (macrophage inflammatory protein 2-alpha, MIP2-α), a chemokine involved in neutrophil and monocyte recruitment.

To gain deeper insights into the biological pathways triggered by TREM-1 activation, we performed gene set enrichment analysis (GSEA) using Gene Ontology (GO) (Figure 5E), Hallmark (Figure 5F), and Reactome (Figure 5G) pathway libraries. Across all three cell types (TANs, *Spp1*^+^ TAMs, and *C1qc*^+^ TAMs) and pathway databases, we consistently observed strong enrichment of interferon and antiviral response pathways. This enrichment aligns with the increased expression of IFN-stimulated genes as seen in the DEG analysis.

A recent review by Meyer et al.^37^ highlights how IFN signaling in myeloid cells enhances anti-tumor immunity by inducing a pro-inflammatory phenotype, characterized among others by increased expression of CD80, CD40, and major histocompatibility complex class II (MHC class II) on macrophages, thereby promoting T cell activation and proliferation.

In addition to IFN signaling, Hallmark and Reactome pathway analysis revealed increased TNF-α signaling via nuclear factor κB (NF-κB) and IL6-JAK-STAT3 pathway, which is supported by our *in vitro* findings where TREM-1L stimulation induced TNF-α and IL-6 production in human monocytes (Figure 2F). Furthermore, we visualized the expression of *Tnfa, Il1b*, and *Il6* in TANs and TAMs following TREM-1 activation *in vivo* (Figures S6A and S6B). Mechanistically, it is known that TREM-1 binding promotes NF-κB activation through the adapter proteins DAP12 and CARD9.^38^ Moreover, pathways related to IL-2 signaling were enriched, suggesting enhanced T cell proliferation and activation (Figures 5F and 5G).

Collectively, these results indicate that TREM-1 activation specifically targets TANs and TAMs and induces changes in their transcriptome toward an immunostimulatory and anti-tumorigenic phenotype. This reprogramming is characterized by the induction of IFN-stimulated genes that lead to increased antigen presentation, as well as to T cell recruitment and activation.

### Cell-cell communication analysis identifies immunity-promoting interactions induced by TREM-1 activation

**Figure 6:**
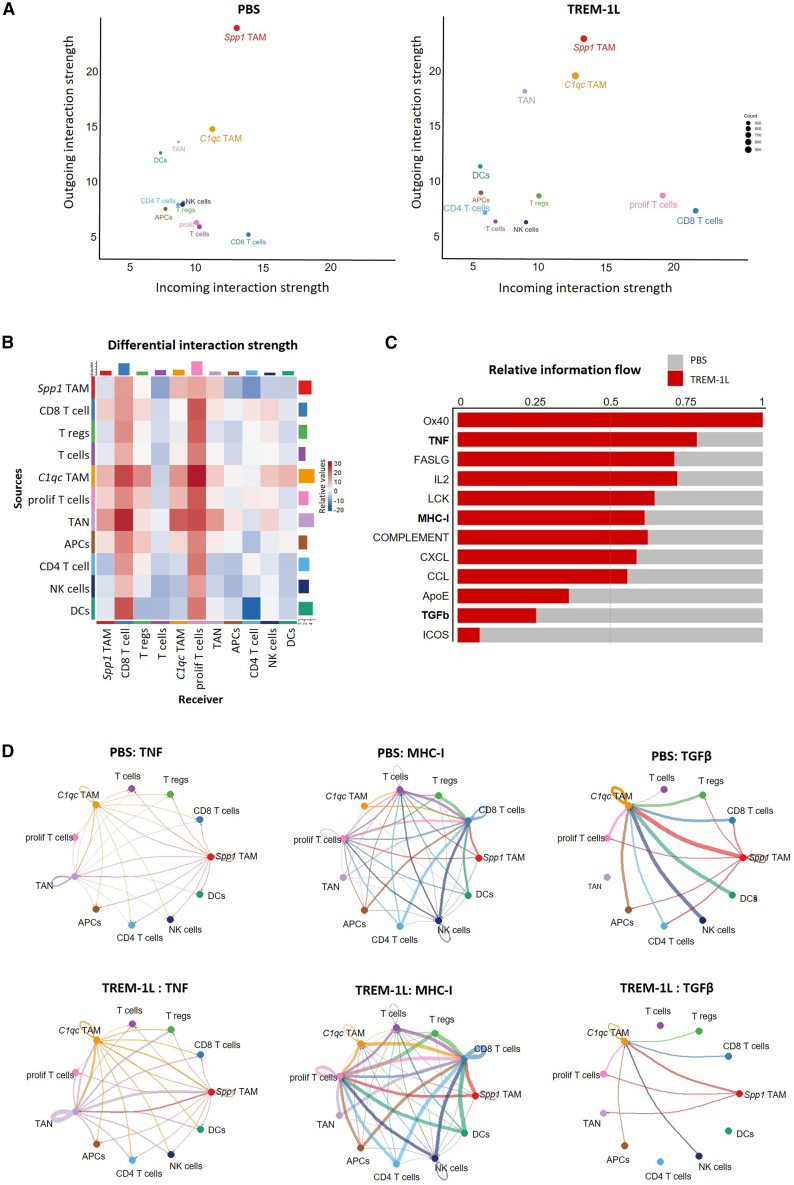
TREM-1 activation enhances immunity-promoting cell-cell communication (A) Incoming and outgoing interaction strength split by treatment. (B) Heatmap showing differential interaction strength following TREM-1L treatment. (C) Relative information flow of signaling pathways. (D) Circle plots representing communication among all cell clusters through representative signaling pathways (TNF, MHC class I, and TGF-β).

The probability of cell-to-cell communication was analyzed by integrating the gene expression data with preexisting information on receptor-ligand interactions using the CellChat package.^39^ To identify the significant changes in the communication among cell populations, we computed the differential strength of cellular interactions (Figure 6A). Notably, CD8^+^ T cells and proliferating T cells received more signals, showing an incoming interaction strength that is almost doubled in TREM-1L-treated tumors. Moreover, signaling by myeloid cells was increased. In particular, *C1qc*^+^ TAMs, *Spp1*^+^ TAMs, and TANs showed higher outgoing interaction strength upon TREM 1L treatment. The heatmap (Figure 6B) illustrates the increased interaction (shown in red) between myeloid cells and CD8^+^ T cells as well as proliferating T cells in the TREM-1L-treated tumors. This indicates that TREM-1 activation not only alters the myeloid cell transcriptome but also significantly affects intercellular communication and signaling between myeloid cells and immuno-stimulatory lymphocytes.

We further assessed the nature of the signaling and examined the information flow of individual signaling pathways (Figures S7A and S7B). Altered signaling pathways are plotted as representatives in a stacked bar plot (Figure 6C). In line with our observations so far, the signaling pathways involved in effective immune responses and cytotoxicity, such as Ox-40, TNF, FASLG, IL2, LCK, MHC-I, the complement system, and various chemokines (CXCL and CCL), were predominant following TREM-1 activation. On the other hand, we observed a higher prevalence of pathways linked to anti-inflammatory immune responses, such as TGF-β or ICOS, in the PBS-treated control tumors (Figure 6C).

In addition, we compared the cell-cell communication for some pathways of interest that were strongly induced upon TREM-1 activation (TNF, MHC class I, and TGF-β), represented in circle plots split between treatment groups (Figure 6D). TNF-α is expressed by TANs and TAMs, and signal interaction is increased in most cell types upon TREM-1 activation. This finding is consistent with our *in vitro* experiments, where we showed that TREM-1L triggers TNF-α production in TREM-1-positive monocytes (Figure 2F). Next, we observed a strong increase in signaling through MHC class I). MHC-I molecules are expressed on all nucleated cells, presenting epitopes to the T cell receptors (TCRs) of CD8^+^ T cells, which is essential for the killing of virus-infected or tumor cells. The upregulation in signaling through the MHC-I pathway may result from an increased CD8^+^ T cell infiltration due to TREM-1L-induced chemokine production by myeloid cells. In contrast, TGF-β signaling is predominant in the PBS-treated tumor, contributing to an immunosuppressive TME that promotes tumor growth and metastasis.^40^ Our data show that signaling through TGF-β is reduced by TREM-1 activation, which further contributes to the reduced immunosuppression in the TME.

Altogether, our results indicate that TREM-1 activation reprograms tumor-associated myeloid cells from an immunosuppressive to a pro-inflammatory phenotype, promoting anti-tumor immune responses.

### TREM-1 expression in human PDAC samples

**Figure 7:**
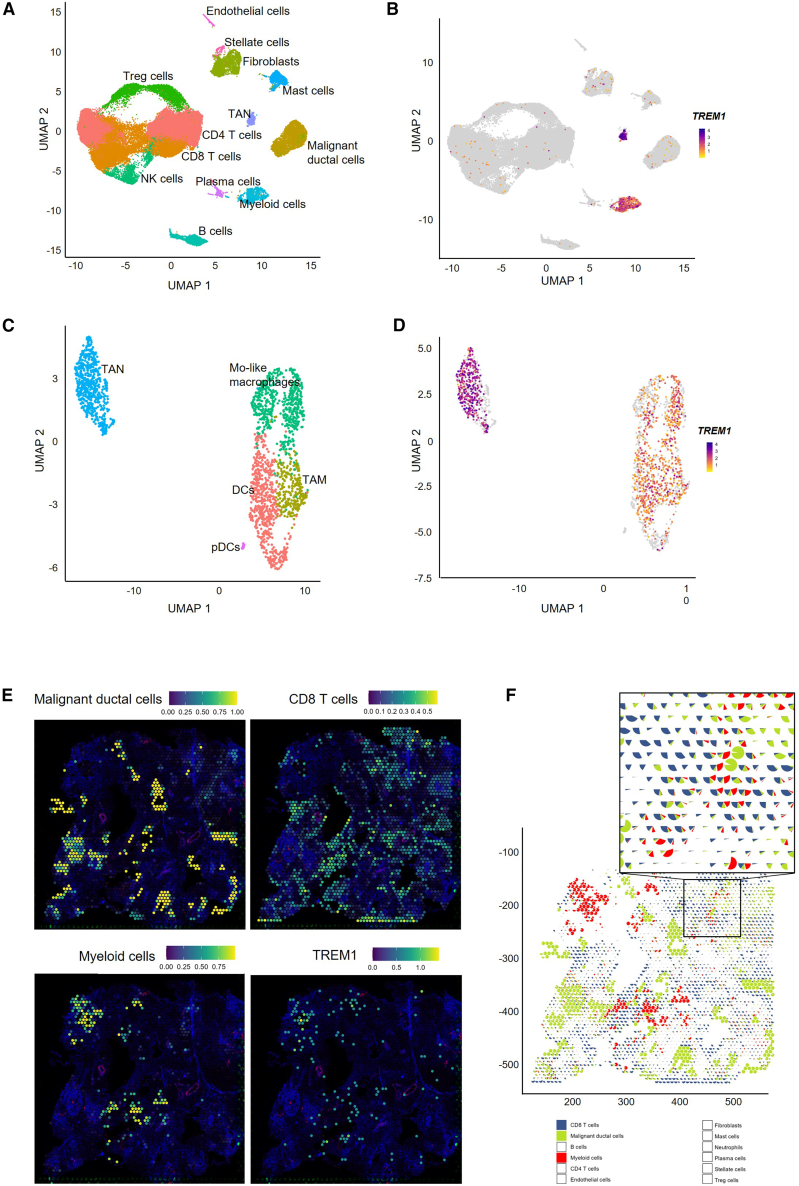
TREM-1 is expressed on TANs and TAMs in human PDAC (A) UMAP plot showing cell clustering and cluster identification in human PDAC samples (*n* = 13). (B) Feature plot showing TREM1 expression. (C) UMAP plot showing the myeloid subclusters and cluster identification. (D) Feature plot showing TREM-1 expression in myeloid subclusters. (E) Spatial feature plots after integration with scRNA-seq gene expression signatures showing malignant ductal cells, CD8^+^ T cells, myeloid cells, and TREM1 expression. (F) Spatial scatter pie chart of a representative sample with zoom-in showing malignant ductal cells, CD8^+^ T cells, and myeloid cells.

To assess the translational potential of our findings, we performed scRNA-seq and spatial transcriptomics analysis on human PDAC tissue. The scRNA-seq data from 13 patients was analyzed using the Seurat package in R. Quality control and filtering resulted in a set of 49,197 cells and using UMAP dimensionality reduction we identified 13 cell clusters, with multiple immune cell types (Figure 7A). The identified clusters are malignant ductal cells, myeloid cells, NK cells, TAN, CD8^+^ T cells, Treg cells, CD4^+^ T cells, plasma cells, B cells, endothelial cells, fibroblasts, and stellate cells. Assessing TREM1 expression in these cells shows that *TREM1* is highly and exclusively expressed in myeloid cells (Figure 7B). To identify the specific cell types that express *TREM1*, we subclustered the myeloid cells and identified five subclusters, namely TANs, TAMs, DCs, monocyte-like macrophages (Mo-like macrophages), and pDCs (Figure 7C). *TREM1* expression levels were highest in neutrophils and macrophages (Figures 7D and S8A). The percentage of *TREM1*^+^ cells was highest in TAM (app. 66%), slightly higher than in the neutrophils (60%), monocyte-like macrophages (50%), and DCs (50%) (Figure S8B).

The direct effects of TREM-1 activation on the human PDAC TME need to be validated in clinical trials involving patients treated with TREM-1L. To support this, we examined the expression of proinflammatory cytokines previously shown to be induced upon TREM-1L treatment *in vivo* (IL-6, IL-1β, and TNF) along with CXCL8, IL-10, and TGF-β1, which are commonly upregulated in the PDAC TME (Figures S9A and S9B). This analysis confirmed the presence of these cytokines in human PDAC samples. Furthermore, feature plots generated from all cells (Figure S9A) and the myeloid subcluster specifically (Figure S9B) confirm the important role of myeloid and neutrophils in cytokine production and cellular signaling. Next, we performed Visium spatial gene expression (10× Genomics) analysis on human PDAC tissues to investigate the spatial distribution of *TREM1* expression. The PDAC morphology is highly disorganized and comprises a broad spectrum of neoplasms, which can present as dysfunctional glands, small clusters, or single tumor cells dispersed within a dense stromal matrix. This diversity is illustrated in the three representative images, which show each an H&E-stained whole-slide section (Figures S10A), a magnified view of a tumor region (Figure S10B), and stroma (Figure S10C).^41^ To further characterize the TME, CD45 immunohistochemistry was used to label immune cells within PDAC (Figure S10D). These staining techniques also visualize the inter- and intratumoral heterogeneity. To resolve the cellular composition of the TME, we integrated the scRNA-seq data with spatial transcriptomics using Seurat-anchor-based integration. This enabled the identification of multiple cell types namely malignant ductal cells, myeloid cells, TANs, CD8^+^ T cells, CD4^+^ T cells, plasma cells, B cells, endothelial cells, fibroblasts, stellate cells, and mast cells. The spatial distribution of these cell types revealed tumor regions spread across the tissue sections and surrounded by many CD8^+^ T cells (Figures 7E and S11A). Myeloid cells, a key component of human PDAC microenvironment, were found in close proximity to both CD8^+^ T cells and malignant ductal cells. TREM1 expression was observed within a subset of myeloid cells, as indicated by the spatial overlap between TREM1 signal and myeloid cell locations (Figure 7E).

To investigate the cellular composition within each spatial detection spot, we employed spatial scatter pie charts to visualize the relative proportions of each cell type per spot. Fibroblasts appeared to be generally abundant across samples (data not shown), while myeloid cells, malignant ductal cells, and CD8^+^ T cells were frequently co-localized in several regions as indicated by composite pie charts displaying two or three of these cell types within the same detection spot, indicating their close spatial proximity (Figures 7F and S11B). To validate these findings, we examined the spatial expression patterns of several marker genes (Figure S11C). We used keratin expression (*KRT7*, *KRT8*, and *KRT19)* to visualize the tumor area and *CD68* to mark myeloid cells. We also assessed CD16 (*FCGR3A*), which is commonly used to identify cytotoxic innate immune cells, such as neutrophils, non-classical monocytes, and CD56^dim^ CD16^+^ NK cells. Finally, we assessed *TREM1* expression in relation to these markers, confirming its co-localization with the myeloid markers *CD68* and *CD15.* The TREM1-positive spots were frequently located in close spatial proximity to CD8^+^ T cells and tumor regions.

The scRNA-seq analysis from our human PDAC samples confirmed what we observed in a publicly available dataset, as well as in the mouse Pan02 model, namely that TREM1 is most abundant on myeloid cells and, more specifically, on macrophages and neutrophils. However, spatial transcriptomics allowed us to specifically map TREM1 expression and identify its cellular localization and proximity to the CD8^+^ T cells and tumor regions. Altogether, these results suggest that TREM-1 is expressed on neutrophils and macrophages in the TME of human PDAC. Therefore, we hypothesize that activation of myeloid cells through TREM-1 may establish a permissive microenvironment that enables CD8^+^ T cells to exert anti-tumor effector functions. These data provide initial pre-clinical evidence and support TREM-1 as a promising target for therapeutic development.

## Discussion

Despite advances in treatment strategies, the survival outcome for PDAC patients remains dismal. PDAC is characterized by a dense stroma and an immunosuppressive TME that contribute to preventing an effective immune response against the tumor. The myeloid population is the most abundant component of the immune compartment in PDAC. Tumor-associated myeloid cells can have immunosuppressive or immunostimulatory properties. In PDAC, most of the myeloid cells exert a pro-tumorigenic function through the production of immunosuppressive cytokines, such as IL-10, and growth factors, such as TGF-β, known to inhibit CD8^+^ T cell activity.^42^ In addition, TAMs trigger the signaling of immune checkpoints, such as CTLA-4 and PD-1, leading to T cell exhaustion.^43^ Moreover, TAMs can secrete matrix proteins and proteases, leading to stromal degradation and driving metastasis formation.^44^ In addition, it has been shown that TAMs promote chemoresistance and that their depletion correlates with an improved response to chemotherapy in PDAC patients.^45^ Compared to depletion of TAMs, their activation and re-polarization could not only restore their tumoricidal phenotype but also promote a proinflammatory TME in PDAC. In this study, we present TREM-1 activation as an effective treatment strategy to reprogram myeloid cells into a proinflammatory phenotype in PDAC and to promote tumor specific immune responses.

These findings challenge the established notion of TREM-1 as a pro-tumorigenic factor, as highlighted in previous research.^46^^,^^47^^,^^48^^,^^49^^,^^50^^,^^51^^,^^52^^,^^53^ Several studies have demonstrated that TREM-1 can promote tumor growth and progression by enhancing the immunosuppressive characteristics of the TME. For instance, a study by Zhao et al.^47^ found that TREM-1 fosters an immunosuppressive TME in papillary thyroid cancer. Similarly, Ho et al.^48^ reported TREM-1 expression in tumor-associated macrophages to be associated with cancer recurrence and poor survival of patients with NSCLC. They conclude that TREM-1 and the inflammatory response may play an important role in cancer progression. Furthermore, it has been suggested that TREM-1 inhibition reduces pancreatic cancer (PC) tumor growth and extends survival in PC mouse models. However, it is important to note that the models used in the study involved immunocompromised mice with a T cell deficiency. They suggest that silencing the TREM-1-mediated signaling pathway could suppress specific inflammatory responses in these mice.^52^

In contrast, our study reveals a different role for TREM-1 in the context of pancreatic ductal adenocarcinoma (PDAC) in immunocompetent mice. We observed that TREM-1 activation within the PDAC TME led to a significant reduction in tumor growth. This effect was attributed to the induction of a pro-inflammatory state in TAMs and TANs, which in turn promoted antitumor immune responses.

Our findings align with the growing body of evidence highlighting the importance of targeting myeloid cells within the TME to enhance antitumor immunity. Similar to the TREM-1 agonistic antibody PY159, which was shown to activate proinflammatory signaling and enhance T cell responses via myeloid cell reprogramming, our study demonstrates that TREM-1L can also modulate the TME by promoting a shift from an immunosuppressive to an immunostimulatory state. Importantly, while Juric et al. employed an antibody-based approach, our results indicate that ligand-mediated activation can achieve comparable outcomes in the context of pancreatic cancer.^23^ This suggests that different modalities, whether antibody or ligand-based, can effectively target the myeloid cell compartment to foster an environment conducive to antitumor immunity. Furthermore, our approach might offer advantages in specific cancer contexts where antibody penetration or sustained activation is a concern, particularly in dense stromal environments like that of pancreatic cancer. Future research should explore the potential synergies between these approaches and investigate the broader applicability of TREM-1L-based myeloid activation across various tumor types.

The differences in TREM-1’s role may arise from the contrasting outcomes of its activation versus inhibition or absence. Activation of TREM-1 might have a different impact during tumorigenesis compared to its role in established tumors, suggesting that TREM-1 activation could potentially disrupt an already established immunosuppressive TME in PDAC. These context-dependent effects underscore the importance of understanding the specific TME and the stage of cancer development when considering TREM-1 as a therapeutic target. Further research is needed to elucidate these mechanisms and optimize TREM-1-based therapies across different cancer types.

By reanalyzing published scRNA-seq data, we identified TREM1 expression in myeloid cell clusters within human PDAC and observed a higher frequency of TREM1-positive myeloid cells compared to normal pancreatic tissue. Across TCGA cohorts, TREM1 expression in PDAC showed one of the strongest correlations with a classically activated macrophage signature. Consistent with these findings, immunofluorescence analysis of human PDAC samples confirmed that up to 20% of all cells and approximately one-third of macrophages expressed TREM-1. These results highlight the significant presence of TREM-1^+^ myeloid cells in PDAC and support its potential role as a therapeutic target for modulating immune activation.

Activation of TREM-1 in human monocytes induces high production of pro-inflammatory cytokines such as TNF-α and IL-1β. TNF-α has been shown to be a mediator for apoptosis of PDAC cells through primed macrophages^54^ and lymphocytes,^55^ and IL-1β is a crucial player for CD11b^+^CD4^+^ cytotoxic T lymphocyte (CTL)-mediated anti-tumor immune response in lung cancer.^56^ These inflammatory cytokines are likely to contribute to the TREM-1-induced reduction of tumor growth. Remarkably, TREM-1L was more effective than agonistic TREM-1 antibody in both inducing *in vitro* cytokine production and reducing tumor growth *in vivo*.

Our mouse and human PDAC data clearly show that TREM-1 is highest expressed in TANs and TAMs, and the same cell clusters show the most significant qualitative and quantitative changes upon TREM-1 activation. Analysis of differential gene expression revealed an upregulation of IFN-associated genes and other pro-inflammatory mediators indicative of a potent anti-tumor immune response and a shift of TAMs and TANs toward an immunogenic phenotype. Recent studies have shown a beneficial effect of IFNγ on the response of pancreatic cancer to immunotherapies, supporting our findings.^57^^,^^58^

The TREM-1-induced reprogramming of myeloid cells toward an immunostimulatory phenotype enables enhanced antigen processing and presentation and increased CD8^+^ T cell activation, promoting T-cell-mediated immunity. Analysis of intercellular communication showed increased pro-inflammatory signal transduction by myeloid cells to CD8^+^ and proliferating T cells such as through TNF or MHC class I and reduced pro-tumorigenic signals such as through TGF-β. It has been shown that macrophage-derived TNF-α impairs pancreatic cancer cell growth^54^ and increased CD8^+^ T cell infiltration has been associated with greater survival in PDAC.^59^

Using spatial transcriptomics, we mapped the distribution of TREM1-positive myeloid cells in human PDAC samples and identified their co-localization with CD8^+^ T cells and malignant ductal cells within the TME. Our findings suggest that activation of myeloid cells via TREM-1L, accompanied by the induction of a pro-inflammatory phenotype, may reshape the TME to become more permissive to effective CD8^+^ T cell responses. In this context, myeloid cells may play a critical role in facilitating cytotoxic T cell activation and recruitment, thereby enhancing anti-tumor immune response.

Some recent preclinical and clinical studies targeted immunosuppressive TAM to repolarize them into pro-inflammatory macrophages, for example, by blocking CSF-1/CSF-1R signaling. This repolarization has been shown to enhance tumor-specific T cell responses and to prevent tumor progression.^60^ Moreover, agonistic CD40 therapy has also been shown to induce a shift of TAMs toward a more tumoricidal phenotype, leading to short-term beneficial responses in PDAC patients.^61^^,^^62^ Another approach is to target the cancer-cell-derived chemokine CCL2, which recruits CCR2^+^ inflammatory monocytes to the TME, where they become immunosuppressive MDSCs and TAMs.^63^ A clinical trial using the CCR2 inhibitor PF-04136309 in combination with FOLFIRINOX demonstrated favorable clinical responses compared to chemotherapy alone.^64^ However, an increase in tumor-associated CXCR2^+^ neutrophils compensated for the beneficial effects of CCR2^+^ macrophage-targeted therapy; therefore, targeting both myeloid cell types appears to be critical to improve response to chemotherapy.^64^

Overall, our data together with these clinical studies suggest that targeting immunosuppressive myeloid cells represents an attractive therapeutic strategy in PDAC patients. We show that TREM-1 activation reprograms myeloid cells in the TME of PDAC toward an immunostimulatory phenotype, resulting in increased tumor-infiltrating lymphocyte activation and enhanced killing of tumor cells, thereby reducing tumor growth.

## Materials and methods

### Cell culture

Murine Pan02 cells were cultured in complete RPMI-1640 medium (Sigma Aldrich, Buchs, Switzerland; supplemented with 10% FCS, 100 units/mL penicillin, 100 μg/mL streptomycin, 1 mM sodium pyruvate, and 2 mM L-glutamine) for 3 days prior to inoculation.

### Monocyte isolation and activation

For human monocyte stimulation, blood was obtained from healthy volunteers (Interregionale Blutspende SRK, Bern, Switzerland). PBMCs were isolated from the peripheral blood by Ficoll-Paque (GE Healthcare) density gradient centrifugation. Subsequently, monocytes were purified using the EasySep Human Monocyte Enrichment Kit without CD16 Depletion (STEMCELL Technologies). For activation, 1 × 10^5^ monocytes per well were cultured for 24 h in the presence of different stimuli: PGN (3 μg/mL, InvivoGen), human PGLYRP1 (1 μg/mL, R&D Systems) or the combination of PGN and human PGLYRP1, anti-TREM-1 (5 μg/mL, clone #193015, R&D Systems), or isotype control (5 μg/mL, IgG1, clone MOPC-21, BioLegend). After 20 min, a crosslinking secondary antibody (2.5 μg/mL, anti-IgG1, clone RMG1-1, BioLegend) was added to all wells with anti-TREM-1 or isotype control antibodies.

### Mice, tumor inoculation, and *in vivo* studies

C57BL/6 mice were purchased from Janvier Labs (France). Eight- to twelve-week-old age- and sex-matched animals were used for all experiments. All mice were housed in specific pathogen-free conditions in the Central Animal Facility (CAF). Mice were randomly assigned to different treatment groups prior to tumor injection. On day 0, tumors were engrafted by subcutaneous (s.c.) injection of 2 × 10^5^ Pan02 cells onto the left flanks of the mice. Mice were treated with intra-tumoral (i.t.) injection of PBS, anti-TREM-1 antibody (30 μg/mL, clone #174031, R&D Systems), isotype control (30 μg/mL), PGN (1 μg/mL, InvivoGen), PGLYRP1 (0.3 μg/mL, R&D Systems), or the combination of PGN and PGLYRP1. Tumor growth was monitored by measuring two dimensions using a digital caliper. Tumor volume was calculated using the formula V = (length∗width^2^)/2. On day 14 post-tumor inoculation, mice were euthanized, and the tumors were isolated. Tumors were enzymatically digested using the gentleMACS Dissociator and Tumor Dissociation Kit (Miltenyi) according to the manufacturer’s instructions. Cells were filtered through a 70 μm cell strainer before proceeding with the fluorescence-activated cell sorting (FACS) protocol. All animal experiments were in accordance with federal regulations and approved by the Cantonal Veterinary Office (BE23/2022).

### Processing of clinical samples

Human PDAC samples were obtained from the Hirslanden Klinik in Bern, Switzerland, from the surgeries of Prof. Dr. med. Kaspar Z’graggen. Human PDAC samples were collected in accordance with guidelines of the Cantonal Ethics Committee (KEK) in Bern under approved protocols (KEK ID: 2017-02246). All participants provided written general consent. The samples were immediately processed after surgery. Each sample was enzymatically digested using the gentleMACS Dissociator and Tumor Dissociation Kit (Miltenyi) according to the manufacturer’s instructions. The cell suspension was filtered through a 70-μm cell strainer before proceeding with the scRNA-seq protocol.

### Fluorescence-activated cell sorting

Zombie Aqua or UV Fixable Viability dye (Zombie dye, BioLegend, San Diego, CA, USA) was used to discriminate dead cells (Zombie positive). Anti-mouse CD45.2 monoclonal antibody (clone 104, BioLegend) was used to label the immune cells from mouse Pan02 tumors. Anti-human CD45 monoclonal antibody (clone HI30, BioLegend) was used to label the immune cells from the clinically obtained PDAC samples. FACS before scRNA-seq was performed using a Beckman Coulter MoFlo ASTRIOS BSL-2 cell sorter at the FACS Lab, Department of BioMedical Research (DBMR), University of Bern, Bern, Switzerland.

### Flow cytometry

Flow cytometry was used to investigate the cellular composition and activation states in Pan02 tumors following TREM1-L treatment. Tumors were harvested on day 14 post-injection, mechanically dissociated through a 40 μm strainer, and single-cell suspensions were prepared. Dead cells were excluded using Zombie Viability dye (BioLegend). Fc receptors were blocked with anti-CD16/32 (Fc Block) prior to surface staining. For surface staining, cells were incubated with antibodies targeting mouse CD45 (#103126, BioLegend), CD3 (#100236, BioLegend), CD4 (#100526, BioLegend), CD8 (#100750, BioLegend), NK1.1 (#108738, BioLegend), and CD69 (#104508, BioLegend) to identify T cell and NK cell subsets and their activation state. Cells were then fixed and permeabilized using the Foxp3 Fixation/Permeabilization Kit (eBioscience) according to the manufacturer’s instructions. Labeled cells were acquired on a CytoFLEX S (Beckman Coulter) and analyzed using FlowJo software (BD Biosciences).

### Single-cell RNA sequencing: 10× genomics

Samples (*n* = 2 for each condition in the murine experiments) for scRNA-seq were submitted to the NGS platform at the University of Bern. Single-cell cDNAs and libraries were prepared using the Chromium Next GEM Single Cell 3′ v3.1 Kit (10× Genomics) on a Chromium Controller. Sequencing was performed on an Illumina NovaSeq 6000 sequencer with an S2 flow cell.

### scRNA-seq data analysis

The 10× Genomics CellRanger (version 7.1, 10× Genomics Inc., Pleasanton, CA, USA) was used to align raw reads to the pre-built mouse refdata-gex-mm10-2020-A; Mouse reference, mm10 (GENCODE vM23/Ensembl 98) and in human refdata-gex-GRCh38-2020-A; Human reference, GRCh38 (GENCODE v23/Ensembl 98). After alignment, the filtered feature barcode matrices were loaded into R (v4.3.2), and downstream analysis was performed according to the Seurat (v4.3.0) workflow.^65^ Briefly, cells were filtered to include only high-quality cells (as defined by <20% mitochondrial RNA and >1,000 genes per cell). Doublets/multiplets were removed using the DoubletFinder (v2.0.3) package.^66^ Data were normalized and log transformed. Harmony (v1.1.0) was used to account for batch effects.^67^ For cluster annotation, two libraries (*ImGenData*, *MouseRNAseqData*) for the mouse dataset and five libraries (*HumanPrimaryCellAtlasData*, *BlueprintEncodeData*, *NovershternHematopoietic Data*, *MonacoImmuneData*, and *DatabaseImmuneCellExpressionData*) for the human dataset from the SingleR package (v2.0.0)^68^ were used together with the expression of established cell-type markers. Gene ontology was performed using ClueGO, a plug-in of Cytoscape.^69^^,^^70^ CellChat (v2.0.0) was applied for the analysis of ligand-receptor interaction and cellular communication.^39^

### Spatial transcriptomics

Clinical samples from PDAC surgeries were obtained and processed for spatial transcriptomics as follows. The sample was cut in a square shape of 8 × 8 mm and embedded in O.C.T. Compound (Sakura) and stored at −80°C until used. The frozen tissue was sectioned and placed on a library preparation slide, fixed, and stained for nuclei with DAPI (Thermo Fisher) and CD45 (LCA, clone 2B11+PD7/26, Agilent). The subsequent steps including imaging, permeabilization, cDNA synthesis, and library construction were carried out following the 10× Spatial Transcriptomics protocol. The raw sequencing data were aligned to a pre-built reference for human genomes (GRCh38) in Space Ranger (v2.0.0). The subsequent analysis was performed by integrating the corresponding scRNA-seq dataset using the anchor-based integration workflow of Seurat.

### Immunofluorescence

Multiplex immunofluorescence was performed on formalin-fixed paraffin-embedded (FFPE) tissues of PDAC samples using an Opal 3-Plex Manual Detection Kit (NEL810001KT, Akoya Biosciences) according to manufacturer’s instructions. Briefly, sections were deparaffinized and rehydrated. Antigen retrieval was performed using Tris/EDTA buffer pH9 for all slides (Dako, #S2367). Protein blocking was performed using blocking buffer and incubating slides in a humidified chamber for 10 min at room temperature. Then, slides were incubated with primary antibodies against CD68 (1:100 dilution, Dako, #M087601-2) and TREM-1 (1:200 dilution, Abcam, #EPR22060-229) at room temperature. Next, incubation with polymer horseradish peroxidase (HRP) Ms + Rb was performed at room temperature for 10 min. Opal signal was generated by incubating the slides with Opal Fluorophore Working Solution containing fluorophores, Opal 520 and Opal 690, at room temperature for 10 min. Microwave treatment was performed to strip the primary-secondary-HRP complex, allowing introduction of the next primary antibody. For detection of the next target, the protocol was recapitulated at the blocking step. For the last step, slides were counterstained with spectral DAPI reagent for 5 min in the dark at room temperature and mounted with VectaShield Vibrance Antifade Mounting Medium (Vector Laboratories, #H-1700). The images were acquired using a 3DHistech Pannoramic 250 Slide Scanner.

### TCGA data collection and analysis

TCGA gene expression data as well as clinical information from all available cancer cohorts were obtained using GDCRNATools.^71^ Counts were normalized using default parameters of the DESeq2 package.^72^ In the PDAC cohort, gene expression of *TREM1* was correlated with a signature for classically activated (IFNγ + LPS, TNF-α) macrophages^34^ using Pearson correlation. Tumor RNA-seq datasets from all TCGA cohorts were scored for the same macrophage signature and TREM-1 expression using the singscore package.^73^

### Statistical analysis

GraphPad Prism v9.0 was used for statistical analysis. *p* values lower than 0.05 were considered significant (∗*p ≤ 0.05*; *∗∗p ≤ 0.01*; ∗∗∗*p* ≤ 0.001; ∗∗∗∗*p* ≤ 0.0001). Each dot represents an individual sample. All bar graphs show means and SEM values.

## Data availability

The data are available from the corresponding author upon reasonable request.

## Acknowledgments

All animal experiments were performed in accordance with federal regulations and approved by the LANAT Amt für Landwirtschaft und Natur, Bern, Switzerland. Code: 31553 and 34641. All patients included in this study signed informed consent to participate according to study approval KEK 2017-02246. The study was financially supported by Vontobel-Stiftung 1747/2023; Wilhelm Sander-Stiftung 2024.058.1; Foundation for Experimental Biomedicine Zürich, Switzerland; Swiss National Science Foundation 320030_176083; Novartis Foundation for medical-biological research; and the Fondazione San Salvatore. We thank Prof. C. Müller for providing the *Trem1*^−/−^ mice. We thank the Flow Cytometry and Cell Sorting (FCCS) facility of the Department for BioMedical Research (DBMR), especially S. Müller, as well as the Next Generation Sequencing (NGS) Platform of the University of Bern and the ITMP team (Institute of Tissue Medicine and Pathology, University of Bern) for their excellent services.

## Author contributions

M.S. designed the study. M.S. and L.G. wrote the manuscript. I.M., L.B., and S.R. performed the *in vitro* and *in vivo* experiments. L.G., H.H., and S.R. performed the computational analysis and visualization. R.L.M. and D.L. provided intellectual input. K.Z. and M.W. provided clinical and histopathological expertise and access to patient material. F.M. provided bioinformatic expertise. H.H., L.G., and M.S. contributed to the manuscript revisions. M.S. secured funding for this project.

## Declaration of interests

The authors declare no competing interests.

## Supplemental information

Document S1. Figures S1–S11

Document S2. Article plus supplemental information
