# Supplementary material for: TREM-1 as a novel immunotherapeutic target to treat pancreatic ductal adenocarcinoma
Source: Mol Ther Oncol. 2025 Aug 14;33(3):201034. doi: 10.1016/j.omton.2025.201034 (PMC12410479; doi:10.1016/j.omton.2025.201034)
Supplement: Document S1. Figures S1–S11 [file mmc1.pdf]

OMTON, Volume *33*

Supplemental information

TREM-1 as a novel immunotherapeutic target to treat pancreatic ductal adenocarcinoma

Lina Gross, Ivanina Mutisheva, Hanne Hillen, Steve Robatel, Martin Wartenberg, Feiyang Ma, Lukas Bäriswyl, Delphine J. Lee, Robert L. Modlin, Kaspar Z'graggen, and Mirjam Schenk

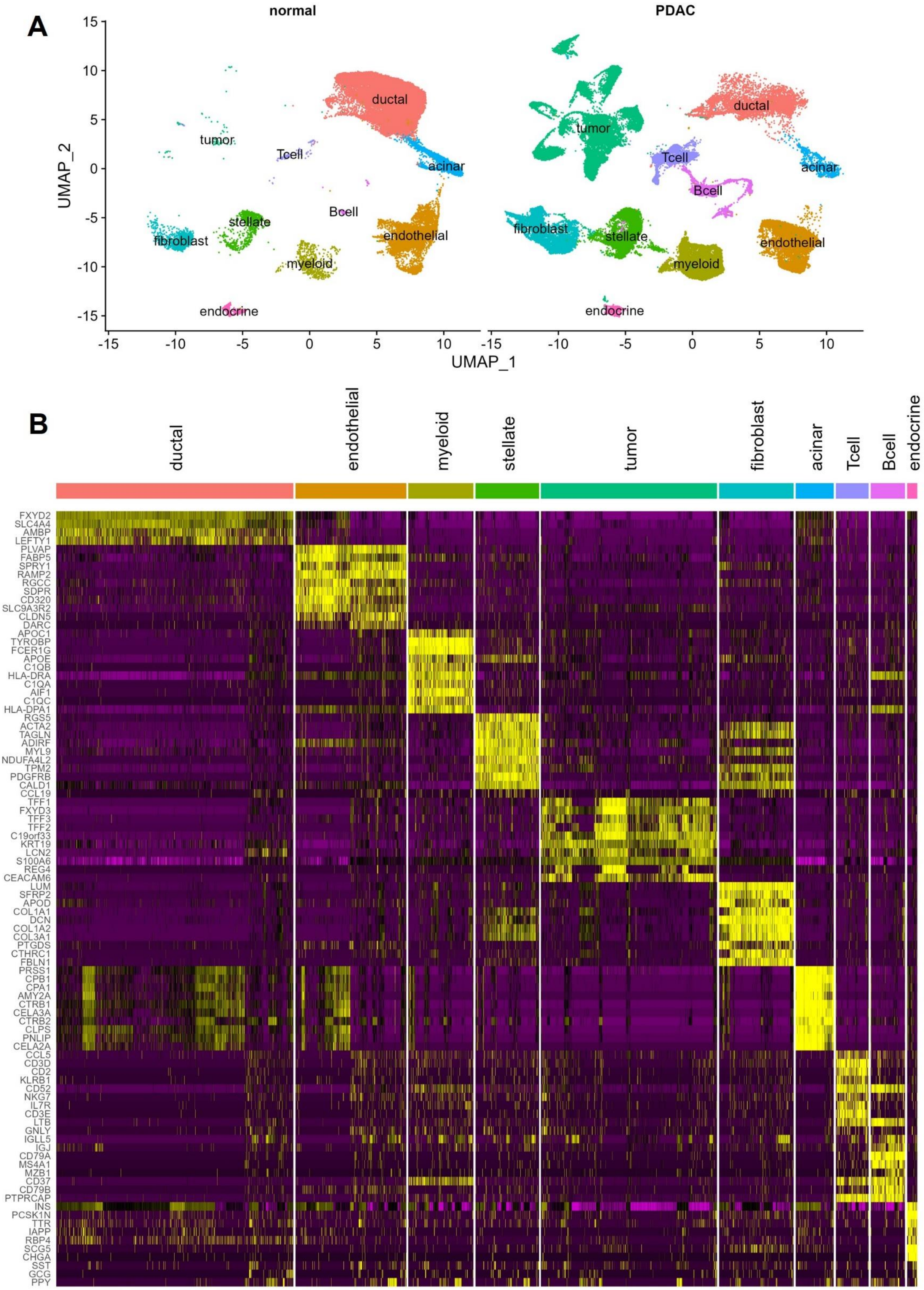

Figure S1. Re-analysis of the PDAC dataset by Peng et al. (2019): Cell type characterization and marker gene expression. A) Umap split by health status of patients. **B**) Heatmap showing expression levels of top 10 cluster markers for main cell types.

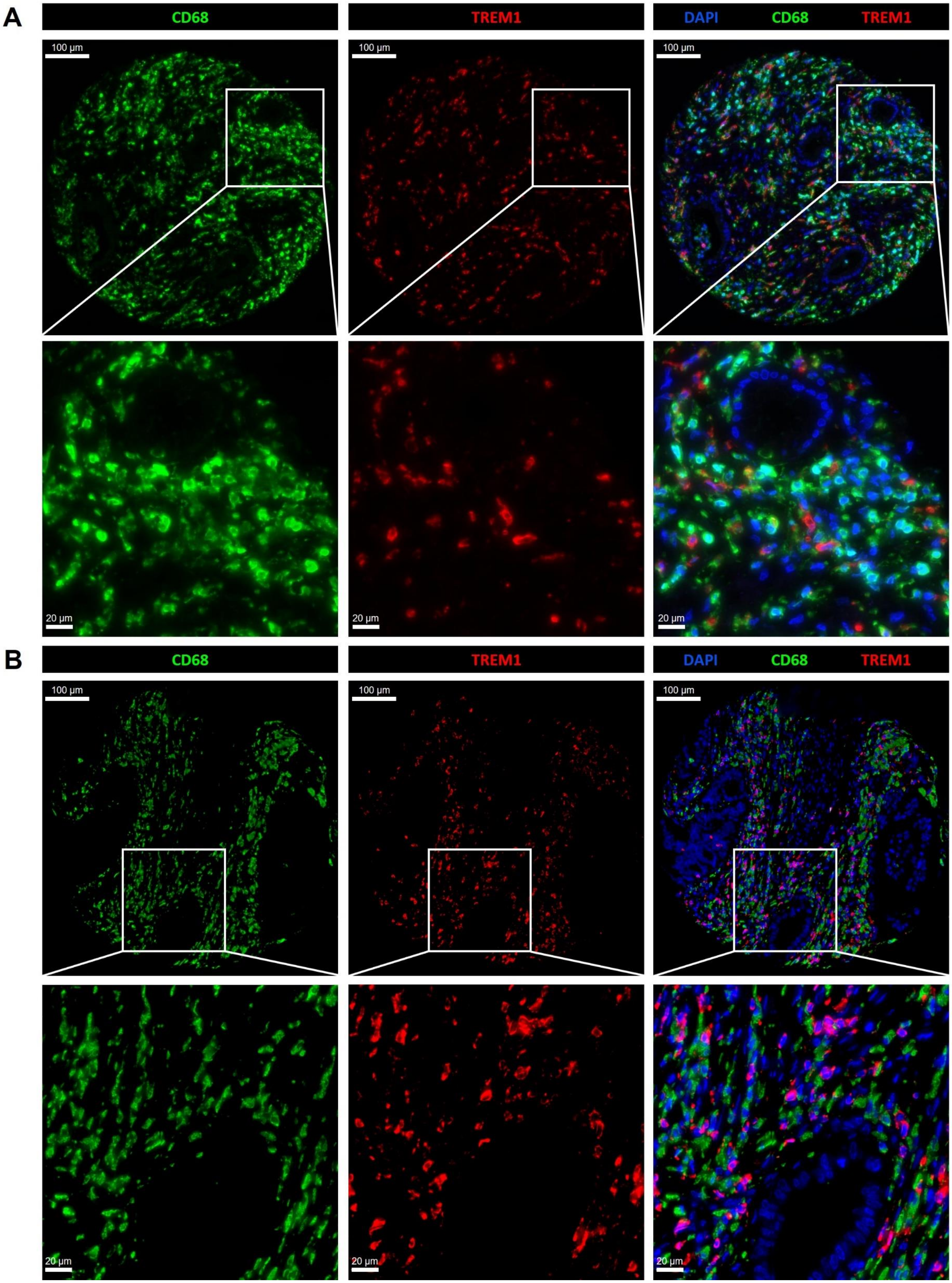

Figure S2. TREM-1⁺ myeloid cells infiltrate human PDAC tissue. (**A, B**) Representative IF images of two independent PDAC samples stained for CD68 and TREM-1, shown with corresponding magnified regions.

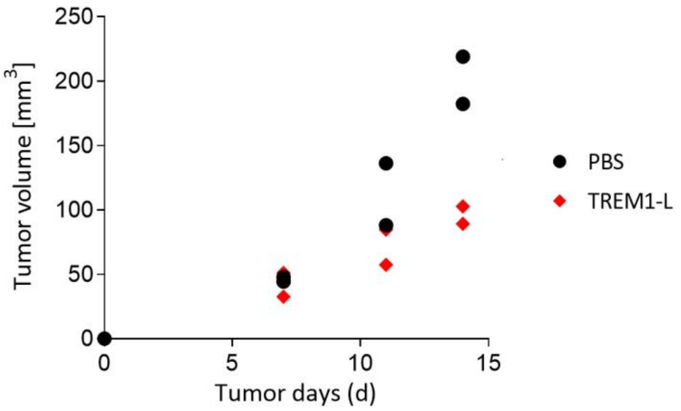

Figure S3. Intra-tumoral treatment with TREM-1L reduced Pan02 tumor growth in mice. Each datapoint represents one mouse used for single-cell RNA sequencing experiments.

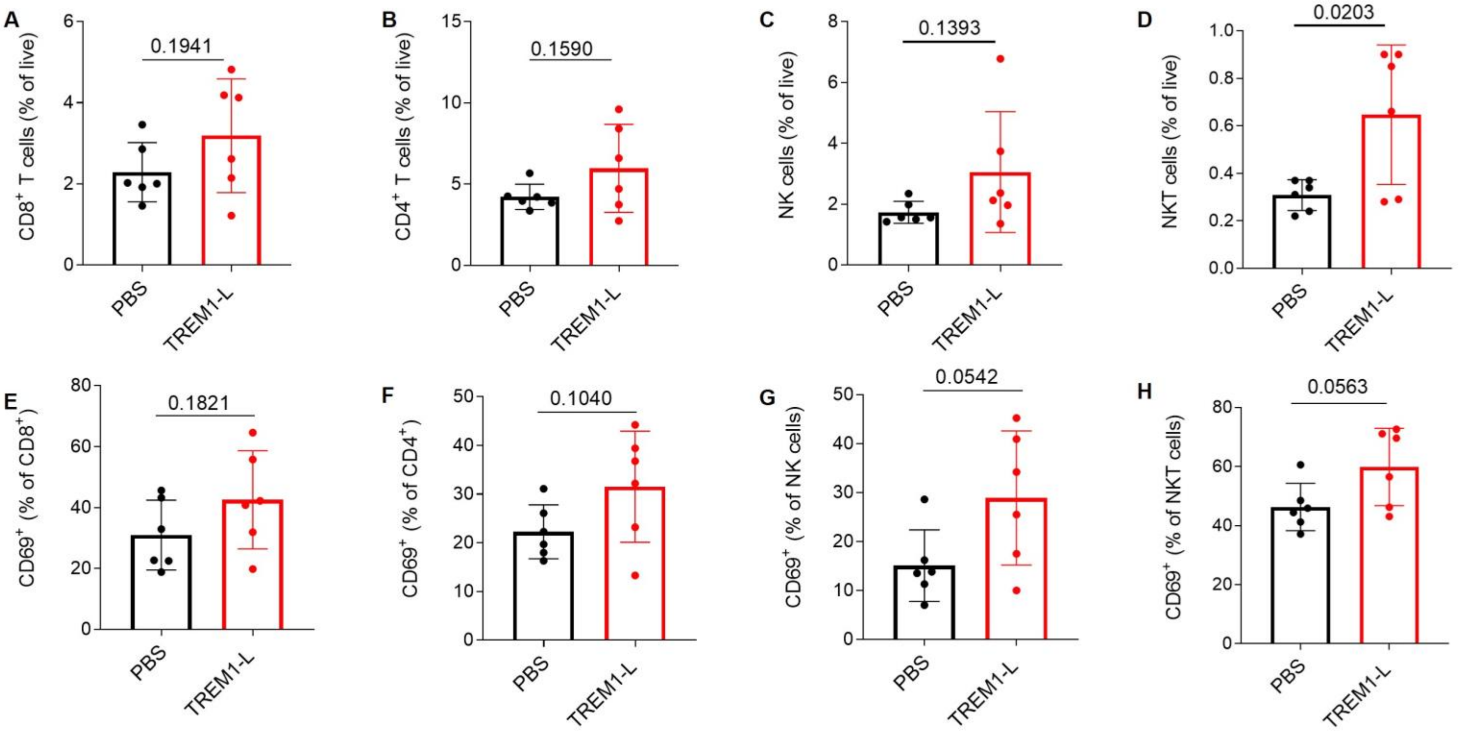

Figure S4. The effect of TREM1-L treatment on cellular level. FACS analysis of (**A**) $CD8^+$ and (**B**) $CD4^+$ T cells, (**C**) NK, and (**D**) NKT cells shown as percentage of live cells, as well as their expression of CD69 as percent of each cell type (**E-H**), (n= 6 per group). Data are represented as mean ± SD using unpaired t-tests.

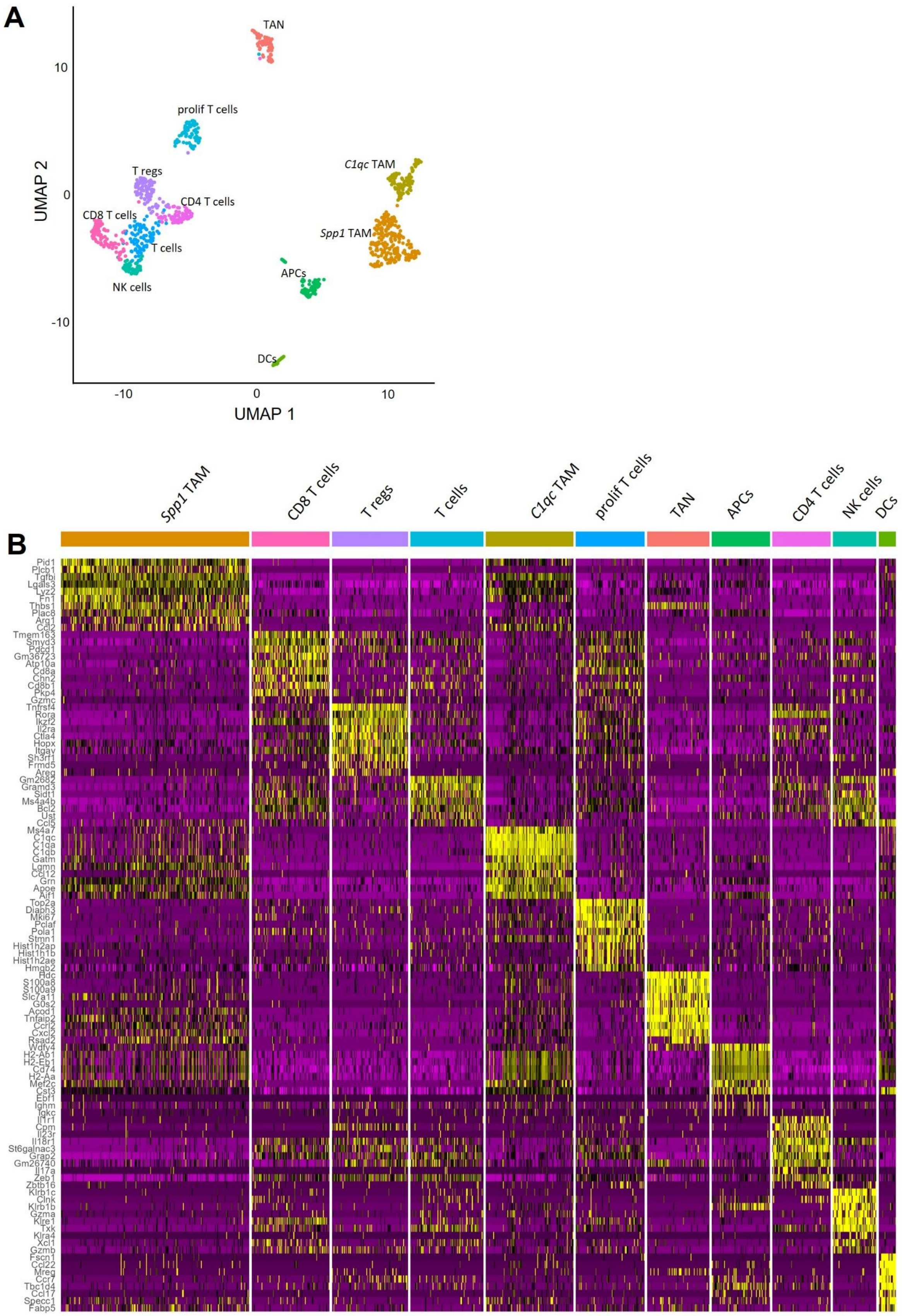

Figure S5. Immune cell clustering and marker gene expression in Pan02 tumors treated with PBS or TREM-1L. (**A**) UMAP visualization of scRNA-seq data from Pan02 tumors treated with PBS or TREM-1L, showing the major immune cell clusters. (**B**) Heatmap displaying the expression profiles of the top 10 marker genes for each identified cluster.

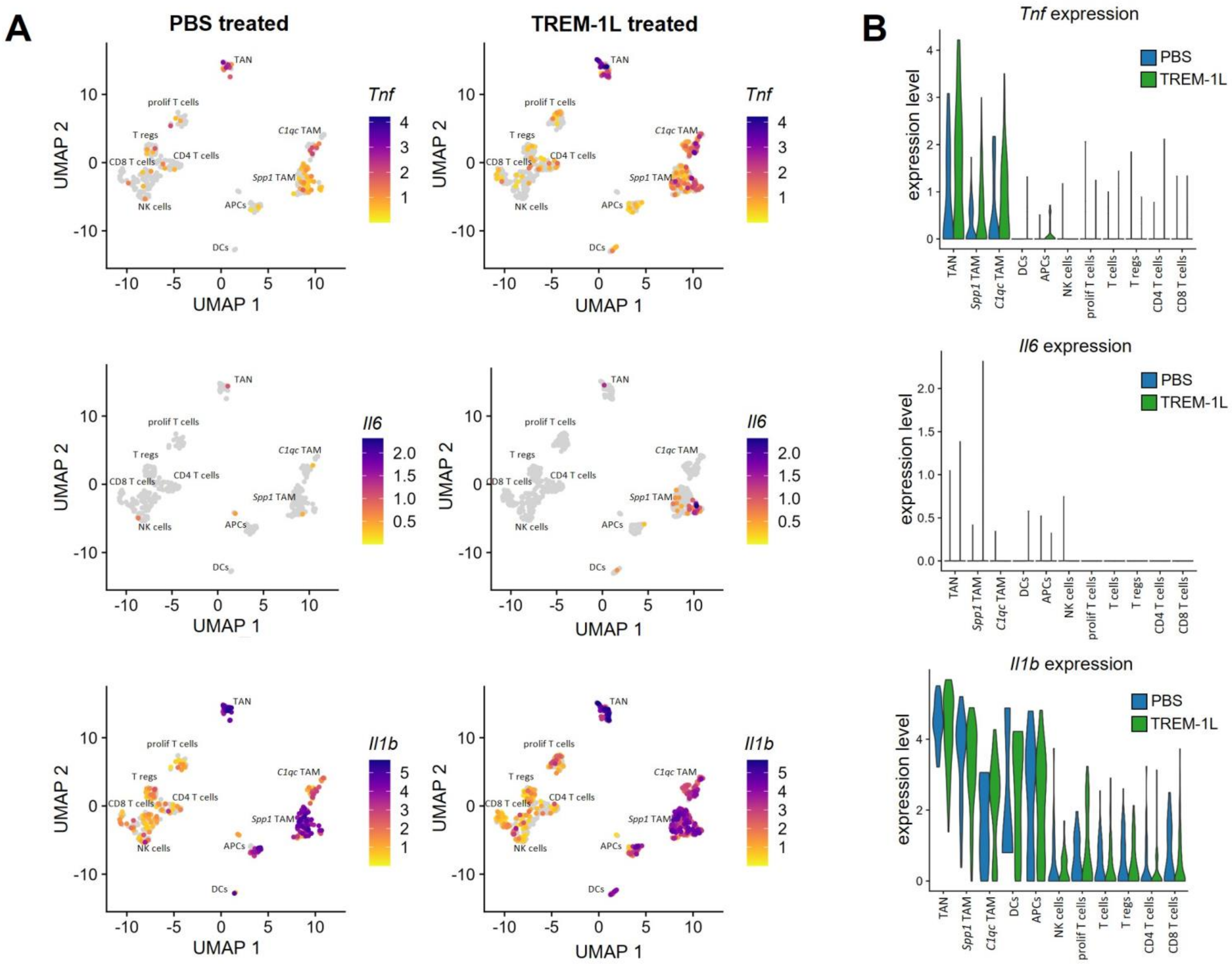

Figure S6. Pro-inflammatory cytokine expression in TAN and TAM following TREM-1 activation in Pan02 Tumors. (**A**) Feature plots visualizing the expression of key pro-inflammatory cytokines in TAN and TAM clusters in Pan02 tumors treated with TREM-1 ligand (TREM-1L) or PBS control. (**B**) Corresponding violin plots showing the distribution and expression levels of these cytokines across the respective cell clusters, highlighting changes upon TREM-1 activation.

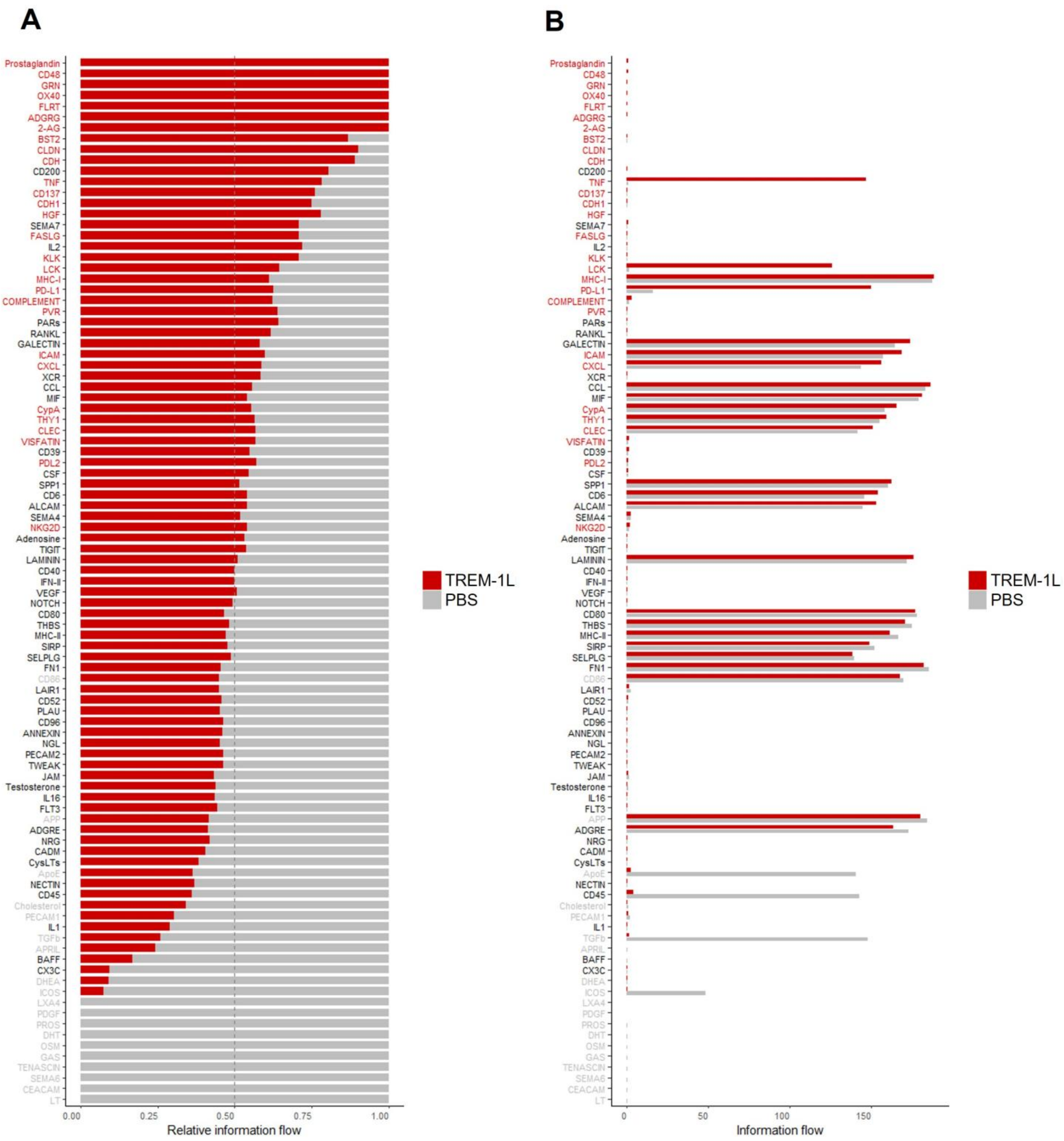

Figure S7. Analysis of information flow of signaling pathways in Pan02 tumors treated with TREM-1L and PBS. (**A**) Relative information flow of all signaling pathways in Pan02 tumors treated with TREM-1L (red) and PBS (grey), calculated using CellChat. (**B**) Absolute information flow of all signaling pathways in Pan02 tumors, highlighting differences between TREM-1L (red) and PBS (grey).

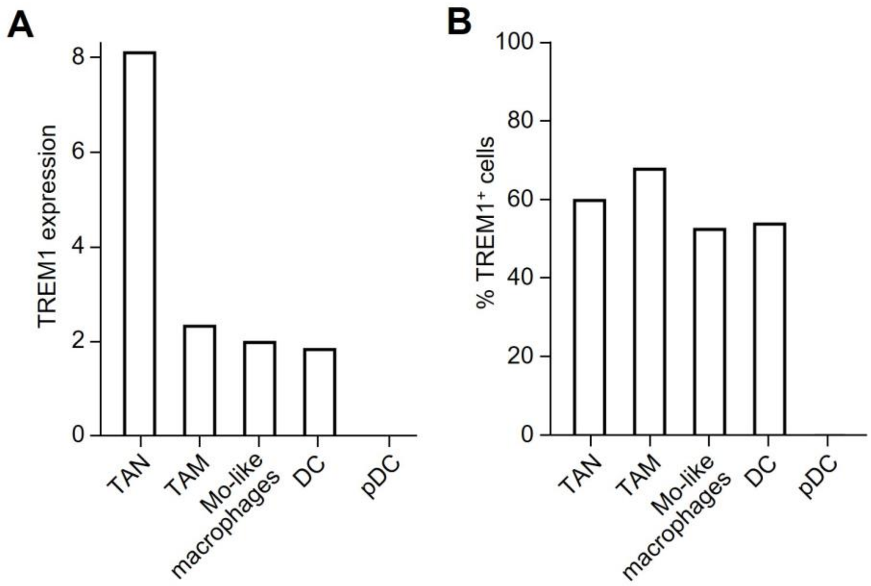

Figure S8. Expression of TREM1 on TAN and TAM subsets in human PDAC. (**A**) Average gene expression levels of TREM1 across various myeloid cell subclusters identified in human PDAC samples. (**B**) Percentage of $TREM1^+$ cells within each myeloid subcluster.

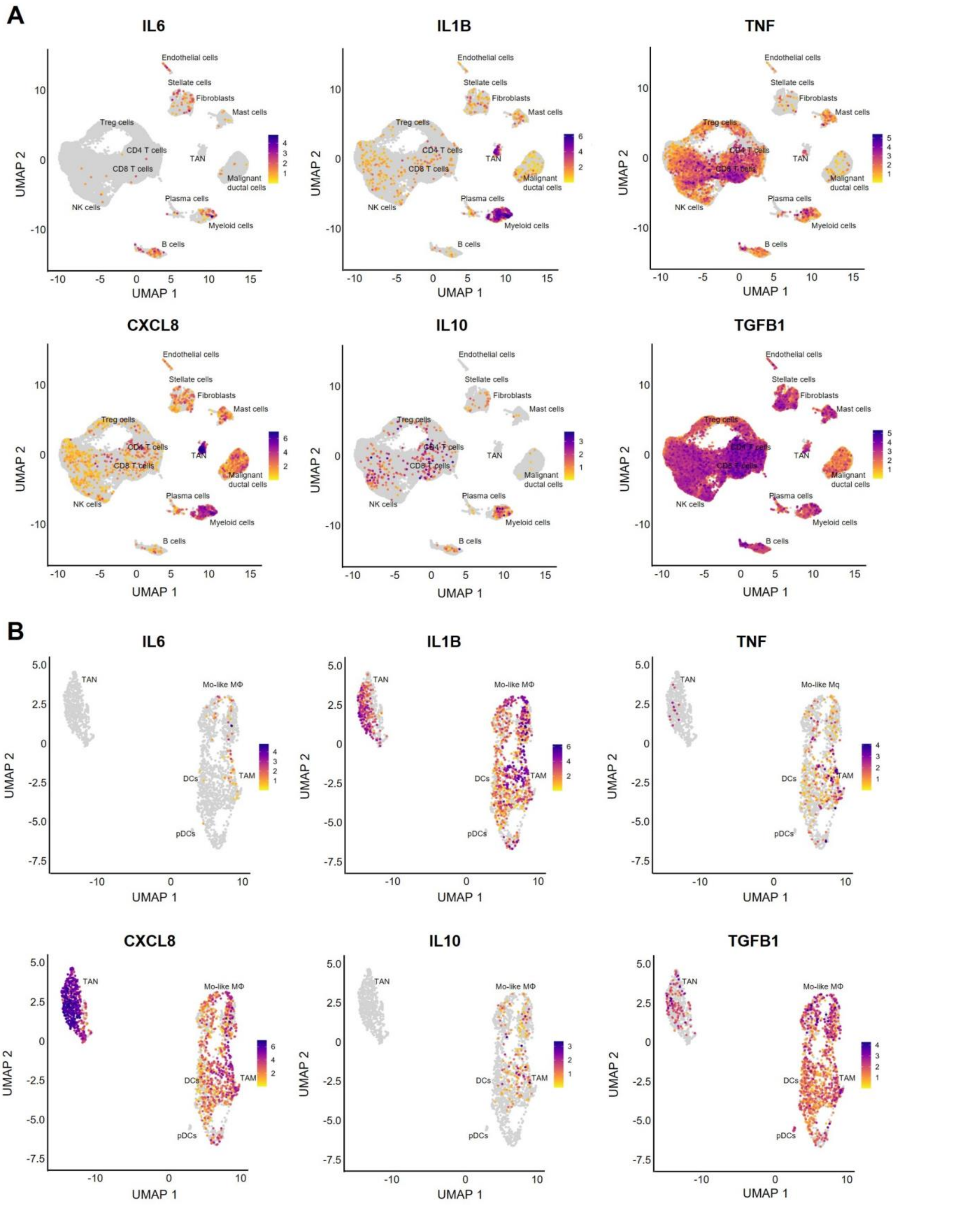

Figure S9. Feature plots showing the expression levels of various cytokines that are shaping the immunosuppressive TME in human PDAC. Panels show the expression of IL6, IL1B, TNF, CXCL8, IL10 and TGFB1 in **(A)** all cell types and **(B)** in specifically in the the myeloid cell subclusters.

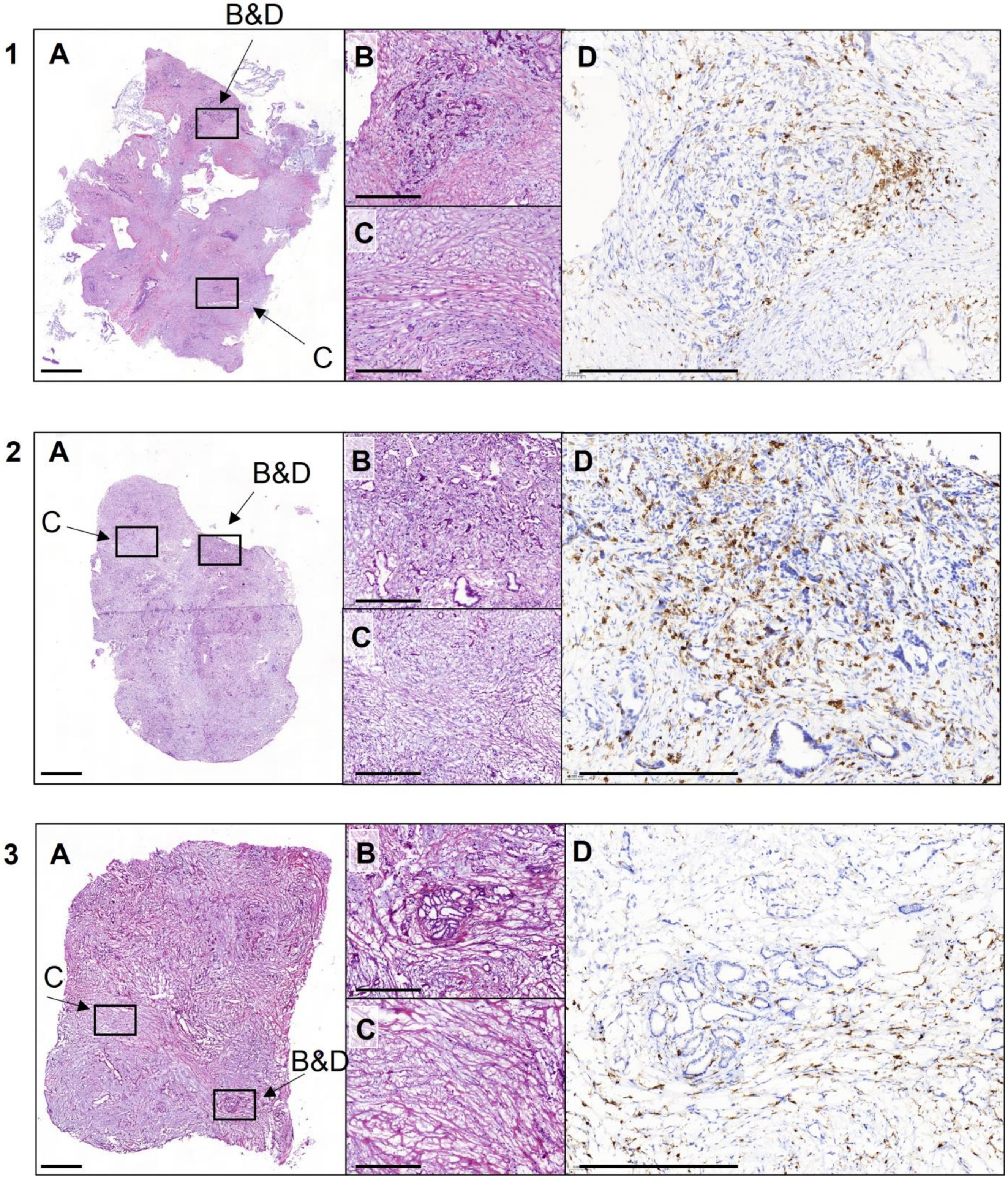

Figure S10. Histological characterization of samples used for spatial transcriptomics analysis. Representative images of tissue sections analyzed by spatial transcriptomics, including (**A–C**) three Hematoxylin and Eosin (H&E) stained sections and (**D**) one CD45 immunohistochemistry (IHC) stained section. (**A**) Overview of the entire tissue sample showing general morphology. (**B**) High-magnification view highlighting tumor cell regions. (**C**) High-magnification view focusing on the TME. (**D**) CD45 IHC staining of the same region shown in (B), marking immune cell infiltration. Scale bars: (**A**) 1 mm and (**B–D**) 200 µm.

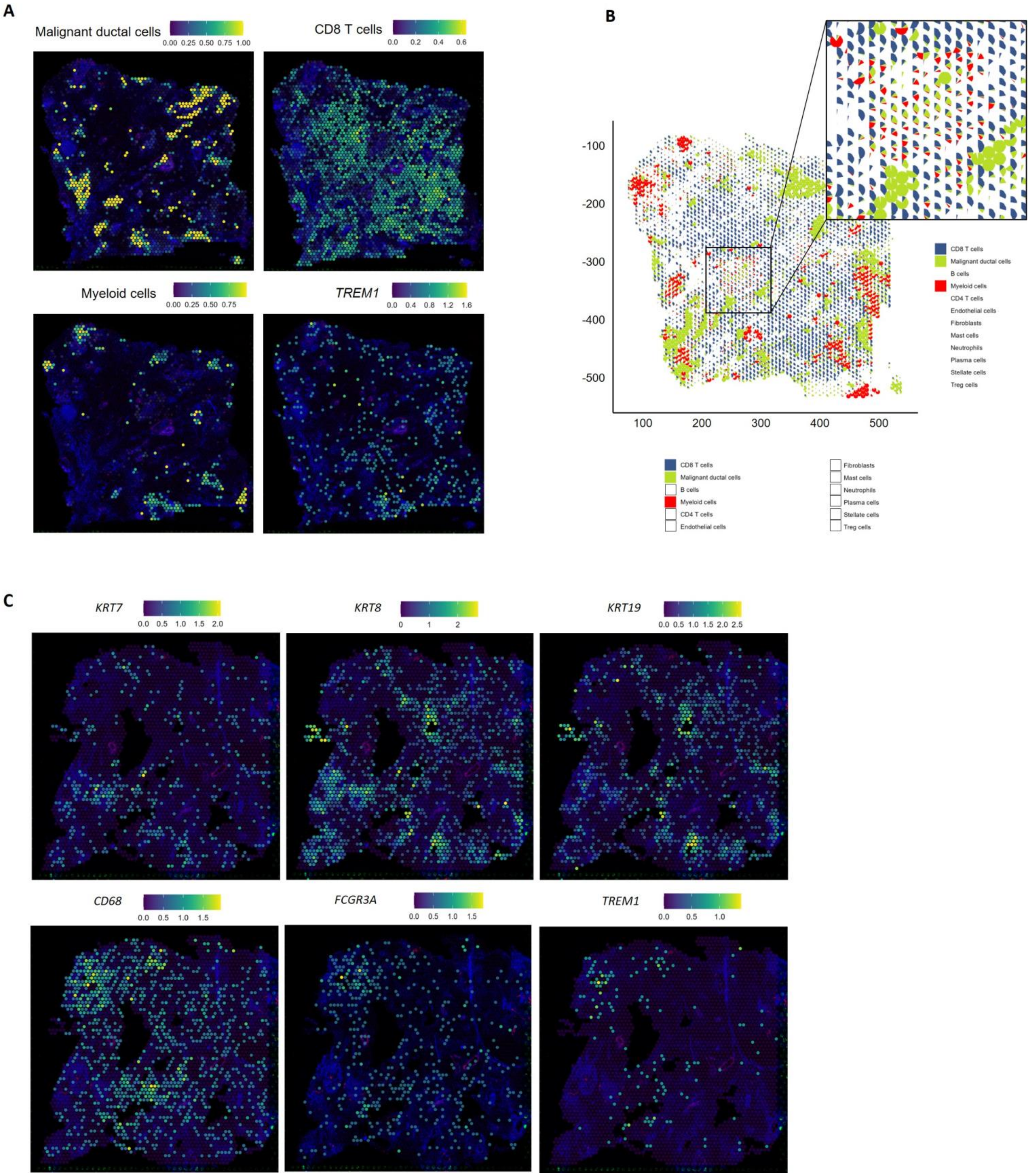

Figure S11. Spatial proximity of myeloid cells and CD8⁺ T cells to malignant ductal cells in tumor tissue. (**A**) Spatial feature plots following scRNA-seq integration illustrating the distribution of malignant ductal cells, CD8⁺ T cells, myeloid cells, and TREM1 expression within the tissue. (**B**) Spatial scatter pie chart from a representative sample (same as in A) with a zoomed-in view highlighting the spatial relationship among malignant ductal cells, CD8⁺ T cells, and myeloid cells. (**C**) Spatial feature plots showing expression patterns of key marker genes, including KRT7, KRT8, KRT19 (epithelial markers), CD68 and FCGR3A (myeloid markers), and TREM1, in the sample corresponding to that shown in Figure 7E.
